# Performance of post-mortem diagnostic tests for tuberculosis in wild ungulates at low and high prevalence assessed using Bayesian latent class models

**DOI:** 10.3389/fvets.2024.1415277

**Published:** 2024-09-25

**Authors:** Beatriz Cardoso, Saúl Jiménez-Ruiz, Alberto Perelló Jiménez, Miguel Nóvoa, João P. V. Santos, Margarida Correia-Neves, Christian Gortázar, Nuno Santos

**Affiliations:** ^1^InBIO Laboratório Associado, CIBIO, Centro de Investigação em Biodiversidade e Recursos Genéticos, Universidade do Porto, Vairão, Portugal; ^2^BIOPOLIS Program in Genomics, Biodiversity and Land Planning, CIBIO, Vairão, Portugal; ^3^SABIO-IREC, Research Group in Health and Biotechnology, Institute for Game and Wildlife Research, University of Castilla-La Mancha, Ciudad Real, Spain; ^4^Departamento de Sanidad Animal, Grupo de Investigación GISAZ, UIC Zoonosis y Enfermedades Emergentes ENZOEM, Universidad de Córdoba, Córdoba, Spain; ^5^Palombar– Associação de Conservação da Natureza e do Património Rural, Vimioso, Portugal; ^6^Life and Health Sciences Research Institute (ICVS), School of Medicine, University of Minho, Braga, Portugal; ^7^ICVS/3B's – PT Government Associate Laboratory, Guimarães, Portugal; ^8^Division of Infectious Diseases, Department of Medicine Solna, Karolinska Institutet, Stockholm, Sweden

**Keywords:** *Mycobacterium tuberculosis* complex, gross pathology, bacteriological culture, real-time PCR, enzyme-linked immunosorbent assay

## Abstract

Animal tuberculosis (TB) is often maintained by multi-host communities, including livestock and wildlife. Quantitative studies of such communities require estimating the true prevalence of TB, correcting the apparent prevalence by the diagnostic sensitivity (Se) and specificity (Sp) of the test. The goal of this study was to lay the foundations for estimating the true prevalence of TB in wild ungulate populations (wild boar and two cervids: red deer and fallow deer). We used Bayesian latent class models to assess the Se and Sp of gross pathology, IS6110 real-time PCR in tissues, bacteriological culture, and P22 indirect ELISA. We analyzed 308 harvested wild ungulates (211 wild boar and 97 cervids: 92 red deer and 5 fallow deer). The Se of bacteriological culture (80.4%, CI_95_ 61.0–96.3%) and gross pathology (87.9%, CI_95_ 69.5–99.9%) was reasonably good in wild boar. These tests showed lower Se in cervids: 60.2% (CI_95_ 38.3–82.3%) for bacteriological culture and 81.5% (CI_95_ 63.6–96.2%) for gross pathology. The Se of the real-time PCR was low (50.7% in wild boar and 53.0% in cervids). These tests showed Sp between 95.2 and 99.1% in both taxa. The P22 ELISA performed reasonably well in wild boar (Se = 71.9%, CI_95_ 59.2–83.4%; Sp = 98.8%, CI_95_ 96.9–99.9%) but lacked Sp in cervids (Se = 77.1%, CI_95_ 62.9–89.7%; Sp = 74.5%, CI_95_ 65.7–83.3%). The real-time PCR in wild boar and cervids and bacteriological culture in cervids tended to show higher Se in low-prevalence populations, possibly due to a higher proportion of early-stage TB lesions. In cervids, the parallel interpretation of gross pathology and bacteriological culture significantly improved the diagnostic performance (Se = 93.1%, CI_95_ 84.7–98.9%; Sp = 92.9%, CI_95_ 86.0–98.3%). Our results allow the estimation of true prevalence from the results of a single diagnostic test applied to harvested wild boar, red deer, and fallow deer, paving the way for more precise quantitative ecological studies of the multi-host TB maintenance community.

## 1 Introduction

Animal tuberculosis (TB), the infection with *Mycobacterium tuberculosis* complex (MTBC), is maintained by multi-host communities, including livestock and wildlife in many regions of the world (1, 2). The concept of the multi-host maintenance community (3) of TB has recently been addressed from a quantitative disease ecology perspective in the Iberian Peninsula (4, 5). These quantitative approaches require estimating the true prevalence of TB in animal populations, correcting the apparent prevalence by the diagnostic sensitivity (Se) and specificity (Sp) of the test employed.

Most wild species playing a role in TB epidemiology in multi-host communities in continental Europe and elsewhere are game species; thus, post-mortem diagnostic tests are convenient methods to estimate the prevalence. Bacteriological culture was traditionally assumed to be the reference test for TB, but it is increasingly considered imperfect. While the Sp of culture tends to be close to perfect, the Se might vary according to the animal species, disease stage, number and type of tissues analyzed, and the analytical protocol employed (6).

The detection of macroscopic lesions (herein gross pathology) is often used as a screening test for TB diagnosis. The Se of meat inspection in cattle has been estimated across studies at 49.9–54.8% (7), while in wild ungulates, although usually performed in the field, it tends to be higher (>70%) (4, 5, 8). The Sp of gross pathology in wildlife is usually estimated at >90% as macroscopic lesions are often characteristic (8). Molecular tests detecting various MTBC genome targets in DNA extracted from tissues have increasingly been used to diagnose TB. A recent meta-analysis found the Se of PCR in cattle to be 69.1–92.3% (7). Likewise, a systematic review estimated the Se of different PCR protocols at 24.6–91.5% in wild boar and 60–100% in deer species (9).

The performance of a diagnostic test was traditionally estimated by comparison with a reference test. However, true reference tests are rarely available, particularly for TB in wildlife. Another approach was to test reference samples of known infection status. Again, these are rarely available in sufficient numbers representative of the populations in the field (10). Latent class models were developed to estimate the performance of diagnostic tests without assuming one of them as a reference test or setting reference infected and non-infected animals (10). Latent class models consider an animal’s actual infection state as unobserved (latent), and the probability of belonging to the infected/non-infected states is derived from the results of different diagnostic tests performed on the same animals, their estimated Se and Sp, and the true prevalence in the population (11). While the parameters of these models were originally estimated by maximum likelihood, Bayesian estimation using Markov Chain Monte Carlo iterations of the model has become the standard approach (10).

The performance of any diagnostic test may depend on epidemiological determinants such as species, age, and particularly the disease stage (6). While the pathogen prevalence in a population *per se* does not affect the performance of the diagnostic tests, unobserved differences in the proportion of disease stages with prevalence might reflect on the diagnostic Se and Sp (6, 12, 13). Nevertheless, the performance of TB diagnostic tests in wildlife has not yet been investigated in different prevalence settings.

This study aimed at laying the foundations for the estimation of the true prevalence of TB in wild ungulate populations from the results of a single post-mortem diagnostic test, correcting the apparent prevalence by the Se and Sp of the test. The specific aims were to (1) evaluate the performance of selected diagnostic tests for TB in harvested wild boar, red deer, and fallow deer, using Bayesian latent class models (BLCMs) to estimate the Se and Sp of gross pathology, IS6110 real-time PCR in tissues, bacteriological culture, and P22 indirect ELISA and (2) compare their Se in populations of low and high prevalence of TB. The study adheres to the STARD-BLCM guidelines in reporting assessments of test accuracy (14).

## 2 Materials and methods

### 2.1 Study sites

**Figure 1:**
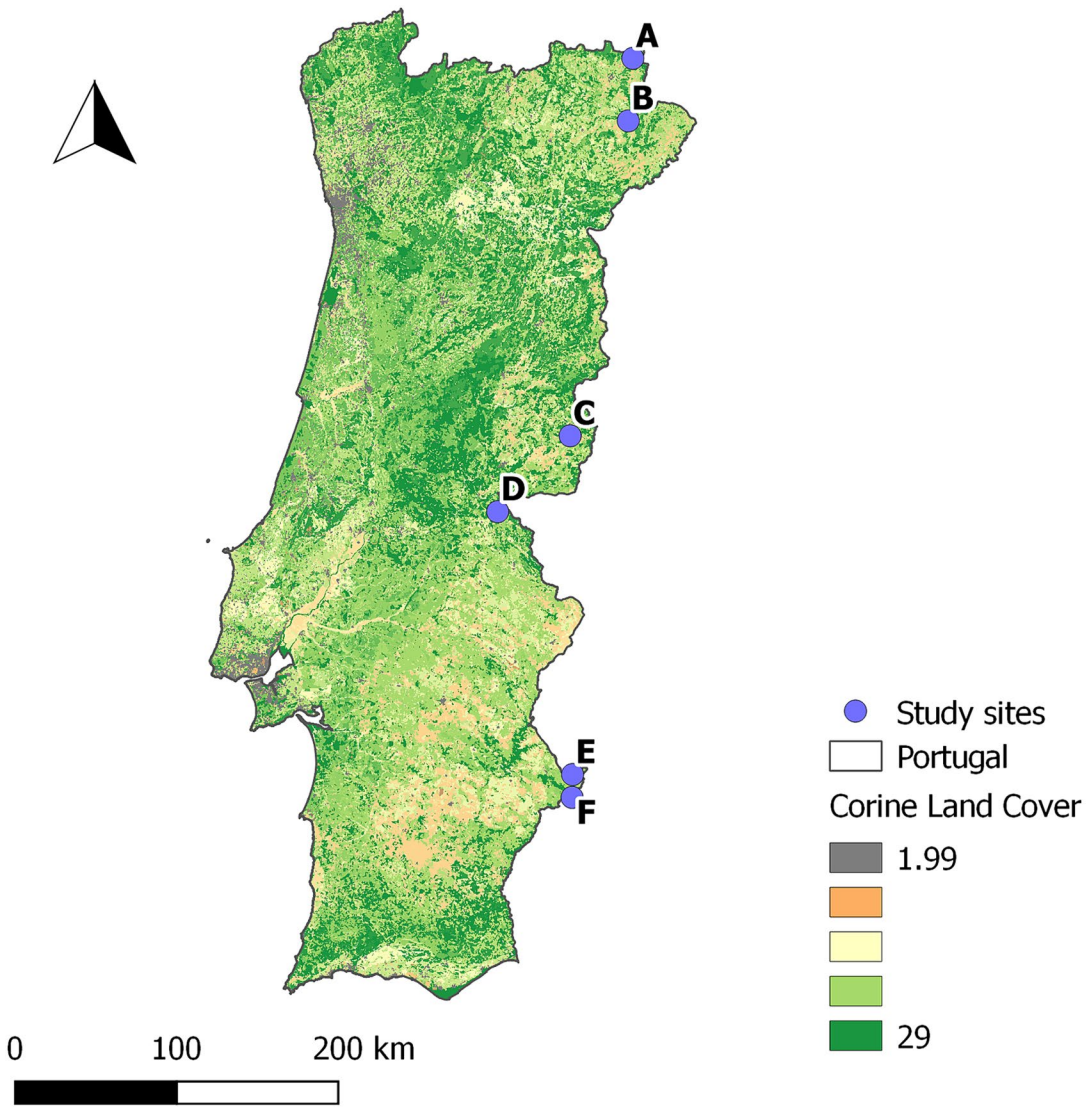
Location of the study sites. Underlying Corine land cover map of Portugal.

This prospective study was conducted in six sites in Portugal (Figure 1), selected to capture the variability in TB epidemiological settings, from areas of known high prevalence in cattle and wild ungulates to areas with no detections in both (15). In all study sites, wild ungulate populations were free-ranging (unfenced) and under low-intensity management, with no provision of food or water. A total of 308 wild ungulates were included in the study: 211 wild boar, 92 red deer, and 5 fallow deer (Table 1).

**Table 1:** Summary of the results of the diagnostic tests per species and study site. Number of ungulates positive for each test in each study site.

Site	Species	Number analyzed	Number positive for animal tuberculosis		
			Gross pathology (Test 1)	IS6110 Real-time PCR (Test 2)	Bacteriological culture (Test 3)
A	Wild boar	90	0	4	0
	Red deer	14	0	0	0
B	Wild boar	37	0	0	1
C	Wild boar	32	0	2	1
	Red deer	10	2	1	1
	Fallow deer	3	1	0	1
D	Wild boar	8	5	3	5
	Red deer	19	4	1	2
	Fallow deer	2	0	0	1
E	Wild boar	29	0	1	0
	Red deer	22	0	0	0
F	Wild boar	15	9	3	10
	Red deer	27	7	6	12
Total	Wild boar	211	14	13	17
	Red deer	92	13	8	15
	Fallow deer	5	1	0	2

### 2.2 Diagnostic tests

#### 2.2.1 Gross pathology (test 1)

The presence of TB-like macroscopical lesions was assessed by the authors (veterinarians with training and experience in *post-mortem* inspection and identification of TB-like lesions) during routine inspections of hunter-harvested wild ungulates. The following lymph nodes were systematically incised and collected in sterile containers and pooled for each animal: submandibular (in wild boar), retropharyngeal (in cervids), tracheobronchial, and mesenteric (in all species). These lymph nodes were selected based on the usual locations of TB lesions in these species (16, 17). Any granulomatous, caseous, or pyogranulomatous lesions were considered as TB-like (8, 16, 17). No animals were killed for the purpose of this study as samples were collected from animals legally harvested by recreational hunters.

#### 2.2.2 Real-time PCR (test 2)

The real-time PCR in tissues started with DNA extraction from an aliquot of pooled tissue homogenates, following a protocol validated for cattle (18), with slight modifications. Approximately 3 g of pooled lymph nodes (submandibular or retropharyngeal, tracheobronchial, and mesenteric) from each animal were homogenized in 4 mL of sterile water, and 1 mL of the homogenate was collected, inactivated with 500 μL of phenol (Sigma-Aldrich, United States), and frozen. DNA was extracted using the DNeasy Blood & Tissue Kit (Qiagen, Germany) with a few modifications. In brief, 500 μL of the homogenized tissue sample was added in a tube containing ~100 μL of 0.1 mm zirconium beads and mechanically lysed in a MiniBead-Beater Homogenizer (Biospec, United States) for 60 s at a speed of 4.8 m/s. After an overnight chemical lysis with 20 μL of proteinase K at 56°C, the mechanical lysis was repeated, 400 μL of a 1:1 mixture of AL buffer and 96% ethanol was added to the lysate and vortexed. 700 μL of the lysate was transferred to a spin column and processed according to the manufacturer’s instructions. DNA elution was carried out using 200 μL of AE buffer supplied in the kit.

*Mycobacterium tuberculosis* complex DNA was amplified following a protocol validated for cattle tissues (19). The real-time PCR protocol targeted a 68 bp fragment of IS6110, specific for MTBC, using the following primers and probes (Thermo Fisher Scientific, United States): 5′-GGTAGCAGACCTCACCTATGTGT-3′ (IS6110_F), 5′-AGGCGTCGGTGACAAAGG-3′ (IS6110_R), and 5′-FAM-CACGTAGGCGAACCC-MGB-NFQ-3′ (IS6110_probe). Real-time PCRs were carried out using the QuantiFast Pathogen PCR IC Kit (Qiagen, Germany), according to the manufacturer’s instructions. A MAX NHS Ester reporter dye-labeled heterologous exogenous internal amplification control (IAC) was used to detect inhibition of the DNA amplification. The cutoff threshold for positivity was set at Ct = 38.7 (19).

#### 2.2.3 Bacteriological culture (test 3)

The bacteriological culture of tissues was performed in a biosecurity level 3 laboratory as previously described (8). The same tissue homogenates that were used to obtain an aliquot for DNA extraction were used for bacteriological culture. In brief, the tissue homogenates were decontaminated in 35 mL of a 0.75% hexadecylpyridinium chloride (Thermo Fisher Scientific, United States) solution for 2 h and centrifuged at 3,500 *g* for 30 min, and three tubes of Coletsos medium (BioRad, United States) were inoculated with 250 μL of the sediment/supernatant interface and incubated at 37°C for 12 weeks. An aliquot of any bacterial growth was collected and suspended in 1 mL of sterile water, inactivated with 500 μL of phenol, and frozen at −20°C (8).

The DNA was extracted from culture suspensions by a standard phenol–chloroform protocol (8) after mechanical lysis with ~100 μL of 0.1 mm zirconium beads (Sigma-Aldrich, United States) in a MiniBead-Beater Homogenizer (Biospec, United States) at 4.8 m/s for 60 s. It was then dissolved in 50 μL of TE buffer and stored at −20°C. The isolates were identified as MTBC based on the amplification of MPB70 or IS6110, following published protocols (18, 19). The MPB70 real-time PCR protocol targeted a 133 bp fragment, specific for MTBC, using the following primers and probes (Eurofins Genomics, Germany): 5′-CTCAATCCGCAAGTAAACC-3′ (MPB70_F), 5′-TCAGCAGTGACGAATTGG-3′ (MPB70_R), and 5′-FAM-CTCAACAGCGGTCAGTACACGGT-BHQ1-3′ (MPB70_probe). Real-time PCRs were carried out using the QuantiFast Pathogen PCR IC Kit (Qiagen, Germany), according to the manufacturer’s instructions.

The cutoff threshold in culture suspensions was set using finite mixture models at Ct = 28.5 for MPB70 and Ct = 22.2 for IS6110 (see Supplementary Figure S1 for details on the method). The dichotomous results (positive/negative) of MPB70 and IS6110 real-time PCRs were interpreted in parallel, that is, an isolate positive to any of the PCR protocols was considered MTBC.

#### 2.2.4 P22 ELISA (test 4)

In a subset of the sampled animals (129 wild boar, 71 red deer, and 4 fallow deer), blood was collected from the endocranial venous sinuses (20, 21) and centrifuged at 2,000 *g* for 10 min to obtain serum samples. The presence of anti-MTBC antibodies was tested using the in-house indirect ELISA protocol described by Thomas et al. (22, 23). In brief, plates were coated with P22 immunopurified protein solution at a concentration of 10 μg/mL in carbonate–bicarbonate buffer overnight at 4°C, and the wells were washed with PBS-0.05% Tween-20 (PBST) and blocked with 5% skimmed milk powder solution in PBS for 1 h at room temperature. Sera were added at a dilution of 1:10 in blocking solution, incubated for 1 h at 37°C, and washed three times with PBST. Protein G horseradish peroxidase (HRP) conjugate (Sigma–Aldrich, United Sates) was added at a concentration of 2 μg/mL in PBS, and the plates were incubated for 1 h at room temperature. After four washes with PBST, the substrate (o-phenylenediamine dihydrochloride) (Sigma-Aldrich, United Sates) was added and incubated for 20 min at room temperature in the darkness. The reaction was stopped with H_2_SO_4_ 3 N, and the optical density was measured at 492 nm.

Positive controls consisted of pooled sera from wild boar or red deer (the same species as the sera to be tested) with MTBC isolation by bacteriological culture. Negative controls used were pooled sera from wild boar or red deer without detected TB-like lesions, negative for isolation of MTBC, and originating from TB-free areas (22, 23).

### 2.3 Latent class models

Given the chronic nature of TB and the biological principles of the diagnostic tests assessed in this study, the definition of “positive” was an animal exposed to MTBC. Hui-Walter models were fitted to the data for three tests across 13 animal populations (24). Individuals were grouped into “populations” by species and study site, resulting in six wild boar, five red deer, and two fallow deer “populations” (Table 1). Test performance was assessed jointly for cervids (red and fallow deer) because of the low number of individuals of the later species included in the study.

Three model chains were run for 10,000 iterations as burn-in, and another 1,000,000 iterations with thin = 10 were used for inference. Model convergence was assessed by visual inspection of autocorrelation, kernel density, trace plots, and potential scale reduction factor (psrf) (25). Models were implemented in R 4.2.1 (26), through RStudio 2022.07.1 (27) using the package “runjags” 2.2.2–1.1 (28). For every model, a leave-one-out sensitivity analysis for the dataset was performed by running the models excluding one animal at a time and comparing the parameter estimates with those obtained using the whole dataset. A sensitivity analysis of the priors was also performed for Model 1 by comparing estimates obtained using priors derived from the bibliography and minimally informative priors.

For those species where no single test yielded a satisfactory sensitivity, the performance of the parallel interpretation of two diagnostic tests was estimated as derived model parameters, according to the equations (29):

$$Se_{\mathrm{parallel}}=1-\left(\left(1-Se_{\mathrm{test}A}\right)\times \left(1-Se_{\mathrm{testB}}\right)\right)$$

$$Sp_{\mathrm{parallel}}=Sp_{\mathrm{testA}}\times Sp_{\mathrm{test}B}$$

The code for all the models is provided in Supplementary material (Codes S1–S3).

#### 2.3.1 Model 1

In Model 1, we estimated the Se and Sp of three diagnostic tests (Test 1: gross pathology; Test 2: amplification of MTBC DNA in tissues; Test 3: bacteriological culture of tissues), with the Se and Sp specified as equal between sites. The Se and Sp of each test were set as different in each of the two taxa considered (wild boar/cervids) as the pathological presentations of TB and the prevalence of other agents causing TB-like lesions could differ between species and influence test performance (8, 16, 30).

Priors based on the bibliography on the performance of diagnostic tests in ungulates (5) were specified in Model 1 (Supplementary Table S1). The median and 95% confidence intervals (CI_95_) reported in the references were used as expert estimates of the underlying beta distribution. The parameters of the beta distributions were estimated in R 4.2.1 (26) with the “betaExpert” function in the package “prevalence” (31). The uncertainty in the estimates extracted from the bibliography was set as high (*p* = 0.1) to broaden the distribution of the parameters, so they should have minimal influence on the posterior distributions. We also ran Model 1 specifying minimally informative Jeffrey’s priors as beta (0.5, 0.5) (32) for both the prevalence and test’s Se and Sp to evaluate the effect of the priors on the posterior distributions.

All possible combinations of pairwise covariances between tests were included in the models, and the one specifying the covariances between gross pathology-bacteriological culture was selected by its deviance information criterion (DIC) (33) (Supplementary Table S2).

#### 2.3.2 Model 2

A variation of the same model was applied to investigate potential differences in test performance in settings with low and high prevalence of TB (Model 2). The data were again grouped into species and populations, with different Se specified by taxa and prevalence (populations with low/high TB prevalence, as estimated by Model 1). The Sp of all tests was set as equal between prevalence settings. The Sp of gross pathology and real-time PCR was set as equal between taxa, while the Sp of bacteriological culture was allowed to vary between taxa, according to the results of Model 1 (Table 2).

**Table 2:** Performance of the diagnostic tests. Summary of the posterior distributions obtained from Model 1 with priors extracted from the bibliography. psrf: potential scale reduction factor.

Species	Diagnostic test	Sensitivity			Specificity		
		Median (CI_95_) (%)	Effective sample size	psrf	Median (CI_95_) (%)	Effective sample size	psrf
Wild boar	Gross pathology	87.90 (69.53–99.85)	30,000	1.0001	98.45 (96.37–99.93)	30,659	1.0000
	Real-time PCR	50.71 (31.98–69.19)	29,634	1.0001	95.19 (92.45–97.47)	30,281	1.0002
	Bacteriological culture	80.37 (60.95–96.33)	29,714	1.0000	99.10 (97.59–99.98)	30,000	1.0001
Cervids	Gross pathology	81.54 (63.55–96.17)	30,375	1.0001	97.41 (91.49–99.99)	30,000	1.0002
	Real-time PCR	52.96 (32.43–74.42)	30,000	1.0002	95.97 (92.64–98.63)	30,000	1.0000
	Bacteriological culture	60.15 (38.30–82.31)	30,000	1.0000	95.85 (91.11–99.71)	31,114	1.0001
	Gross pathology × Bacteriological culture (in parallel)	93.11 (84.69–98.92)	29,655	1.0000	92.88 (85.96–98.26)	30,000	1.0000

#### 2.3.3 Model 3

Another variation of the first model was used to investigate the diagnostic performance of P22 ELISA, together with gross pathology and bacteriological culture (Model 3). The priors for the Se and Sp of gross pathology and bacteriological culture were set according to the results of Model 1 (Supplementary Table S1). The priors for the Se and Sp of P22 ELISA were specified based on Thomas et al. (23) and Hui and Walter (24), following the procedures for estimating the beta distribution parameters from expert data, as described for Model 1. Again, all possible combinations of pairwise covariances between tests were included in the models, and the one specifying the covariance between P22 ELISA-bacteriological cultures was selected by its DIC (Supplementary Table S2).

## 3 Results

Gross lesions were detected in 28 animals (14 wild boar, 13 red deer, and 1 fallow deer), and MTBC was detected by real-time PCR in 21 (13 wild boar and 8 red deer) and by bacteriological culture in 34 (17 wild boar, 15 red deer, and 2 fallow deer) (Table 1; cross-tabulated results in Supplementary Table S3).

### 3.1 Model 1

In the wild boar, gross pathology and bacteriological culture showed the best diagnostic performances. The same tests showed lower Se in cervids, but the parallel interpretation of gross pathology and bacteriological culture greatly improved the diagnostic performance. The real-time PCR showed low Se in both taxa, with reasonable Sp (Table 2).

The correlation between the Se of gross pathology and bacteriological culture was 0.179 (CI_95_–0.081/0.420) in wild boar and − 0.121 (CI_95_–0.359/0.155) in red deer; for Sp, it was 0.014 (CI_95_–0.015/0.057) in wild boar and 0.025 (CI_95_–0.053/0.207) in red deer. The prior specification did not significantly affect the posterior distribution of the parameters, particularly for Sp where all differences were < 5%. The Se showed differences <10% for all parameters except bacteriological culture in wild boar, which was higher when specifying Jeffrey’s priors (91.3%, CI_95_ 70.9–100%), and real-time PCR in cervids, which was lower (40.7%, CI_95_ 20.3–62.8%) (Supplementary Figure S4).

The prevalence estimated across sites varied from 0.4 to 68.1% in wild boar, 2.1 to 45.9% in red deer, and 34.1 to 37.7% in fallow deer, with huge uncertainty in the latter species due to the extremely low sample sizes (Table 3). Model 1 was mostly insensitive to changes in the dataset, particularly for Sp parameters (Supplementary Figure S4).

**Table 3:** Animal tuberculosis prevalence estimated at each study site. Summary of the posterior distributions obtained from Model 1 with priors extracted from the bibliography. psrf: potential scale reduction factor.

Site	Species	Median (CI_95_) (%)	Effective sample size	psrf
A	Wild boar	0.36 (<0.001–2.51)	29,607	1.0000
	Red deer	2.88 (<0.001–14.72)	30,000	1.0000
B	Wild boar	0.85 (<0.001–6.30)	30,000	1.0000
C	Wild boar	1.06 (<0.001–7.78)	29,657	1.0001
	Red deer	19.99 (<0.001–45.40)	30,340	1.0002
	Fallow deer	34.14 (<0.001–77.06)	29,707	1.0000
D	Wild boar	64.92 (31.77–95.73)	30,000	1.0000
	Red deer	13.49 (0.88–33.30)	30,433	1.0003
	Fallow deer	37.73 (<0.001–88.70)	30,000	1.0001
E	Wild boar	0.96 (<0.001–7.16)	30,000	1.0000
	Red deer	2.11 (<0.001–10.39)	29,288	1.0002
F	Wild boar	68.13 (42.22–92.73)	29,500	1.0000
	Red deer	45.88 (23.11–66.56)	29,704	1.0000

### 3.2 Model 2

**Figure 2:**
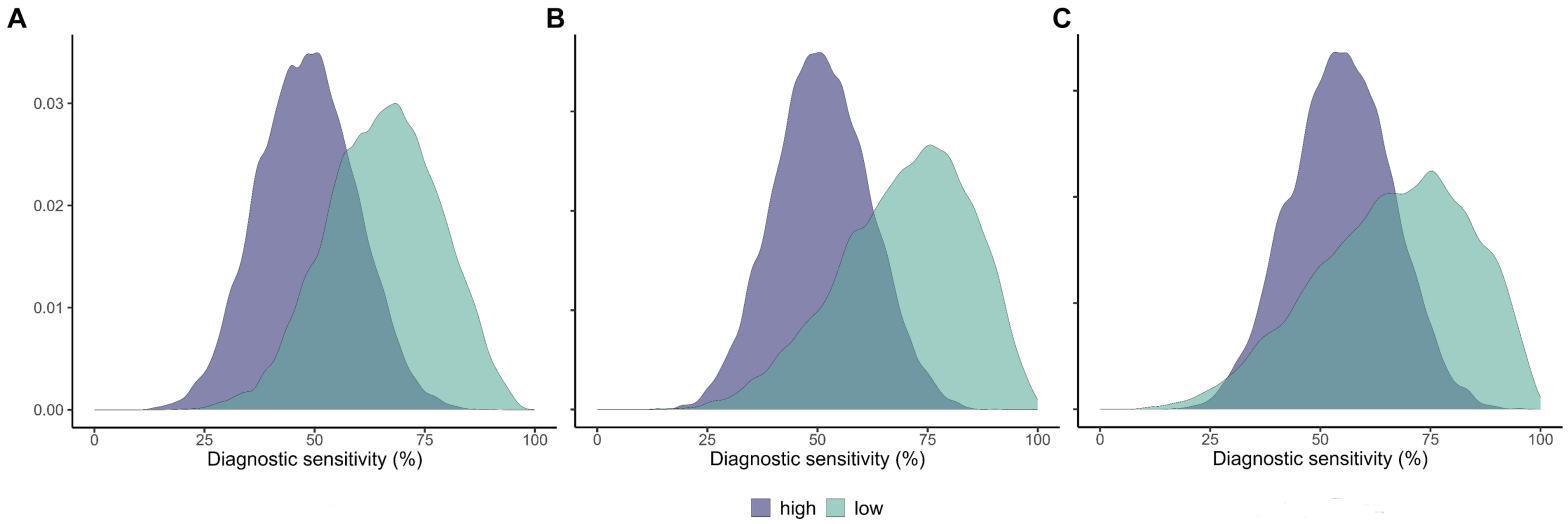
Estimated sensitivity of selected diagnostic tests in populations of low and high prevalence of animal tuberculosis. Posterior distributions of the diagnostic sensitivity of the IS6110 real-time PCR in wild boar **(A)** and cervids **(B)**, and bacteriological culture in cervids **(C)**. High-prevalence populations in magenta and low-prevalence populations in green.

From the results of Model 1, the average prevalence of TB in wild boar was 3.3% (CI_95_ 1.3–8.6%) in the low- (pooled sites A–E) and 68.1% (CI_95_ 42.2–92.7%) at the high-prevalence (site F) groups. In cervids, it was, respectively, 6.2% (CI_95_ 0.1–19.3%; pooled red deer sites A, C, and E) and 32.8% (CI_95_ 12.6–55.7%; pooled red deer sites D and F and fallow deer C–D). Wild boar population D was pooled in the low-prevalence group to allow reasonable uncertainty in the parameter estimates, given it has a small sample (*n* = 8) despite high prevalence. Otherwise, the very low number of positives to any test in the low-prevalence pool led to extremely high uncertainty in the parameter estimates, precluding any meaningful interpretation. Model 2 estimated higher Se for the real-time PCR in wild boar and cervids, and bacteriological culture in cervids, in the low-prevalence populations (Table 4; Figure 2). Model 2 results were robust to changes in the dataset, with slight differences in the Se estimates in the pooled low-prevalence group due to the small number of positive test results (Supplementary Figure S4).

**Table 4:** Performance of the diagnostic tests in settings of low and high prevalence of animal tuberculosis. Summary of the posterior distributions obtained from Model 2 with priors extracted from the bibliography. For all parameters, psrf: potential scale reduction factor <1.0004 and effective sample sizes 28,475-30,742.

Species	Diagnostic test	Median (CI_95_) (%)		
		Sensitivity		Specificity
		Low prevalence	High prevalence	Low and high prevalence
Wild boar	Gross pathology	82.55 (58.21–99.58)	84.74 (65.39–98.47)	98.99 (97.27–99.99)^1,2^
	Real-time PCR	65.77 (41.26–89.26)	48.27 (26.89–70.08)	96.12 (93.90–97.96)^1,2^
	Bacteriological culture	75.38 (48.46–97.64)	76.37 (54.66–94.21)	99.08 (97.50–99.98)^1^
Cervids	Gross pathology	73.87 (49.13–94.65)	82.50 (64.78–96.51)	98.99 (97.27–99.99)^1,2^
	Real-time PCR	71.56 (40.80–96.04)	50.74 (30.10–72.18)	96.12 (93.90–97.96)^1,2^
	Bacteriological culture	67.92 (34.38–96.72)	54.77 (32.53–76.97)	95.62 (90.88–99.39)^1^

^1Estimates shared across TB—low- and high-prevalence populations.^

^2Estimates shared across taxa.^

### 3.3 Model 3

Model 3 estimated the Se of the P22 ELISA to be similar in the wild boar and cervids (71.9–77.0%, respectively). The Sp was 98.8% (CI_95_ 96.9–99.9%) in the wild boar and 74.6% (CI_95_ 65.7–83.3%) in cervids (Table 5). The correlation between the Se of the P22 ELISA and bacteriological culture was – 0.180 (CI_95_–0.310/− 0.031) in wild boar and – 0.234 (CI_95_–0.396/− 0.034) in cervids; for Sp, it was 0.036 (CI_95_–0.017/0.286) in wild boar and 0.011 (CI_95_–0.059/0.113) in cervids. Model 3 was insensitive to changes in the dataset (Supplementary Figure S4).

**Table 5:** Performance of the diagnostic tests. Summary of the posterior distributions obtained from Model 3 with priors extracted from the bibliography (P22 ELISA) and the posterior distributions of Model 1 (gross pathology and bacteriological culture). psrf: potential scale reduction factor <1.0004 and effective sample sizes 29,119-30,636 for all parameters.

Species	Diagnostic test	Median (CI_95_) (%)	
		Sensitivity	Specificity
Wild boar	Gross pathology	87.70 (76.74–95.76)	98.28 (96.77–99.43)
	P22 indirect ELISA	71.88 (59.24–83.43)	98.80 (96.86–99.97)
	Bacteriological culture	84.42 (72.10–95.03)	99.38 (98.45–99.94)
Cervids	Gross pathology	74.18 (61.53–85.41)	97.22 (93.97–99.54)
	P22 indirect ELISA	77.07 (62.91–89.73)	74.47 (65.68–83.29)
	Bacteriological culture	64.07 (51.71–75.92)	99.12 (97.98–99.86)

## 4 Discussion

The evaluation of the diagnostic performance of any test by comparison with a reference test is seldom warranted, as diagnostic tests with perfect performance are exceedingly rare, particularly when applied to wildlife (13). Accordingly, our study shows that no single test yielded 100% Se and Sp for the post-mortem diagnosis of TB in wild ungulates.

Latent class models estimate the diagnostic performance without assuming any of the tests to be perfect and have been increasingly used and endorsed by international regulators (10, 34). Nevertheless, these models were rarely used to investigate TB in wild species [(35), e.g., with Eurasian badgers and (36) with wild boar], where usually the results of a single imperfect diagnostic test are used to estimate the apparent prevalence.

In wild boar, bacteriological culture showed a reasonable performance, with Se = 80.4% (CI_95_ 61.0–96.3%) and Sp = 99.1% (CI_95_ 97.6–99.9%). The estimated Se is higher than that estimated in France using BLCM (42.8%, CI_95_ 19.0–70.6%) (36), which could be related to the fact that only submandibular lymph nodes were cultured in that study, while we cultured pooled submandibular, tracheobronchial, and mesenteric lymph nodes. Gross pathology also performed well in wild boar, with Se = 87.9% (CI_95_ 69.5–99.9%) and Sp = 98.5% (CI_95_ 96.4–99.9%). The location of TB lesions in the wild boar, with a marked preference for submandibular lymph nodes, together with their usually large size, macroscopical appearance, and relatively low proportion of the non-visible lesion’s presentation, might explain this good performance (8, 16, 37, 38).

The real-time PCR targeting IS6110 in tissue homogenates showed poor Se in the wild boar and cervids (Se = 50.7–53.0%). Specificity was relatively high and similar between taxa (95.2–96.0%), even though the target sequence IS6110 can be present in non-MTBC mycobacteria (19). Other studies reported higher sensitivity for conventional and real-time PCR protocols in wild boar (62.5–66.7%) (8, 36). The real-time PCR protocol used in this study was previously validated in cattle by comparison with bacteriological culture, assumed as the reference test (19). That study reported a slightly lower Sp in cattle (93.7%, CI_95_ 91.5–95.3%) than we estimated in wild ungulates. Nevertheless, the Se in cattle was much higher (96.5%, CI_95_ 93.9–98.2%) than we estimated in wild ungulates (19). Modifications in the DNA extraction protocol could explain these differences, as we inactivated the tissue homogenates with phenol and the mechanical lysis step employed lower movements per second (4.8 m/s for 1 min). Differences in the type of lesions could also be involved. Cattle should show a higher proportion of early-type, poorly organized lesions due to the constant removal of infected animals detected by the eradication programs (39, 40). On the contrary, wild ungulates tend to show advanced-stage, mineralized, or pyogranulomatous lesions, often with thick fibrous capsules (16, 17), which could impair the recovery of MTBC DNA.

The real-time PCR tended to be more sensitive in wild boar and cervid populations of low TB prevalence than in those of high prevalence. In cervids, the same result arose with bacteriological culture and the contrary with gross pathology (Table 4; Figure 2). We hypothesize that advanced-stage, necrotic, and calcified paucibacillary lesions (16, 38) might be more common in high-prevalence populations, hindering MTBC DNA recovery. On the contrary, a higher proportion of early non-calcified lesions might be found in low TB prevalence settings, as suggested for red deer infected with *Mycobacterium caprae* (41). In addition, it was shown in other species that early-stage TB lesions tend to have higher mycobacterial load (40). This hypothesis could explain our results, as the recovery of MTBC DNA is likely more difficult from calcified and paucibacillary lesions, frequent in our tissue samples where most infected animals originated from high TB prevalence populations.

Gross pathology and bacteriological culture showed significantly lower estimated Se in cervids than in wild boar (Table 2), as also shown in France (37). Regarding gross pathology, the lower Se estimated in cervids could be due to a higher proportion of non-visible lesions’ presentation than in wild boar (17). Tuberculosis lesions in cervids, being usually paucibacillary pyogranulomatous with a thick fibrous capsule (17, 37), could also impair the detection of MTBC by bacteriological culture, as shown in cattle (42). A mismatch between red deer that test positive for gross pathology or culture has previously been suggested (43).

Model 1 yielded estimates of the Sp of the gross pathology and real-time PCR similar between taxa but also a tendency for lower Sp of bacteriological culture in cervids (95.9%, CI_95_ 91.1–99.7% *vs* 99.1%, CI_95_ 97.6–99.9%). We hypothesize this potentially lower Sp of real-time PCR in cervids could be due to different types of bacterial isolates from TB-like lesions causing non-specific amplification of the IS6110 amplicon.

No test was suitable as a single TB diagnostic method in cervids, but the combination of gross pathology and bacteriological culture in parallel yielded an estimated Se = 93.1% (CI_95_ 84.7–98.9%) and Sp = 92.9% (CI_95_ 86.0–98.3%). This improvement in the diagnostic performance further evidences a complementarity between these diagnostic methods. Advanced-stage and conspicuous lesions are more easily detected by gross pathology, but they might prove more difficult for MTBC detection. It was shown in experimentally infected red deer that MTBC load in tissues tended to decrease with time and was inversely related to the size of the lesions (44). However, the inverse was observed in naturally infected fallow deer (45), so the relationship between diagnostic performance, lesions stage, and MTBC load remains hypothetical.

The in-house indirect ELISA using P22 protein complex as antigen confirmed the good Sp in wild boar previously reported (98.8%, CI_95_ 96.9–99.9%) (22), although with slightly lower Se (71.9%, CI_95_ 59.2–83.4% vs. 84.1%; CI_95_ 79.3–88.2%). On the contrary, in cervids, we confirmed the good Se previously reported (70.1%, CI_95_ 63.6–76.0%) (23) but estimated lower Sp (74.5%, CI_95_ 65.7–83.3% vs. 99.0%, CI_95_ 96.5–99.8%). The suboptimal performance of the P22 ELISA in naturally infected red deer has been suggested (46).

The sensitivity analysis showed the estimates from all models to be mostly insensitive to changes in the dataset. Prior specification using minimally informative Jeffrey’s priors non-significantly affected the results of Model 1, particularly the Se which was lower for all tests in cervids and real-time PCR in wild boar (Supplementary Figure S4). Regarding Model 2, some bias might have been introduced by including wild boar population D in the low-prevalence group. If not, the low number of positives in this pool led to parameter estimates with high uncertainty, precluding any meaningful interpretation. This decision might have diluted the differences in test prevalence between low- and high-prevalence groups, which should be interpreted as minimum estimates.

Our results should be representative of the harvested proportion of the overall populations, as in most hunting events all the harvested ungulates were sampled. Only in the larger or poorly organized events, an opportunistic sample of the harvested ungulates was obtained. Nevertheless, the overall representativeness of our sample toward the larger population of ungulates might have been affected by harvesting bias (47). These results are based on the analysis of a limited pool of tissues from every animal sampled. While the selection of the tissues was based on knowledge of the preferential location of TB lesions in these species (16, 17), higher sensitivity could be achieved by analyzing more tissues. Nevertheless, the potential effect of pooling the tissues to analyze is transversal to the whole sample and should not affect the conclusions.

A central assumption of BLCM is the independence of the tests, conditional on the true infection status of the individuals (10). As this assumption is rarely justified, the covariances between the results of the diagnostic tests must be modeled; otherwise, biased estimates might be obtained (48). Following the approach by Cheung et al. (10), we compared models including all possible pairwise combinations of covariances between tests, and the most supported model assumed no covariance between any of them. Nevertheless, we selected for inference the model specifying the covariances between gross pathology-bacteriological culture, which was almost equally supported (ΔDIC = 1.3) (Supplementary Table S2).

## 5 Conclusion

In this study, we estimate the performance of four post-mortem diagnostic tests in naturally infected wild boar, red deer, and fallow deer and compare three of those between populations at low and high TB prevalence. Bacteriological culture and gross pathology were the best-performing tests in wild boar. All tests showed poor Se in cervids, but the parallel interpretation of gross pathology and bacteriological culture significantly improved the diagnostic performance. Real-time PCR in wild boar and cervids and bacteriological culture in the later taxa tended to have higher Se in settings with a low prevalence of TB. We hypothesize that these differences were due to the differential proportion of late-stage lesions, where the detection of MTBC was likely impaired due to the external fibrous capsules and the low number of mycobacteria, according to TB prevalence. Our results allow for the estimation of true prevalence from the results of a single diagnostic test or combination of tests applied to harvested wild boar, red deer, and fallow deer, paving the way for more precise insight from quantitative ecological studies of the multi-host TB maintenance community.

## Data Availability

The original contributions presented in the study are included in the article/Supplementary material, further inquiries can be directed to the corresponding author/s.
