# Supplementary material for: Performance of post-mortem diagnostic tests for tuberculosis in wild ungulates at low and high prevalence assessed using Bayesian latent class models
Source: Front Vet Sci. 2024 Sep 25;11:1415277. doi: 10.3389/fvets.2024.1415277 (PMC11461446; doi:10.3389/fvets.2024.1415277)

Supplementary material

Performance of post-mortem diagnostic tests for tuberculosis in wild ungulates at low and high prevalence assessed by Bayesian latent class models

Beatriz Cardoso[a,b,c], Saúl Jiménez-Ruiz[d], Alberto Perelló Jiménez[c], Miguel Nóvoa[e], João P. V. Santos[a,b,c,e], Margarida Correia-Neves[f,g,h], Christian Gortázar[c], Nuno Santos[a,b]

[a] CIBIO, Centro de Investigação em Biodiversidade e Recursos Genéticos, InBIO Laboratório Associado, Universidade do Porto, Vairão, Portugal

[b] BIOPOLIS Program in Genomics, Biodiversity and Land Planning, CIBIO, Vairão, Portugal

[c] SABIO-IREC. Research Group in Health and Biotechnology, Institute for Game and Wildlife Research, University of Castilla-La Mancha, Ciudad Real, Spain

[d] GISAZ-ENZOEM. Animal Health and Zoonoses Research Group, Competitive Research Unit on Zoonoses and Emerging Diseases, University of Cordoba, Cordoba, Spain

[e] Palombar– Associação de Conservação da Natureza e do Património Rural, Vimioso, Portugal

[f] Life and Health Sciences Research Institute (ICVS), School of Medicine, University of Minho, Braga, Portugal

[g] ICVS/3B's – PT Government Associate Laboratory, Braga/Guimarães, Portugal

[h] Division of Infectious Diseases, Department of Medicine Solna, Karolinska Institutet, Stockholm, Sweden

Fig S1. Finite mixture models of the cutoff threshold for the MPB70 and IS6110 real-time PCR in isolates.

Table S1. Beta distributions for the sensitivity and specificity of the diagnostic tests and their bibliographical support.

Table S2. Comparison of models including all combinations of pairwise covariances between diagnostic tests.

Code S1. Code for the selected Hui-Walter model including the covariance between Test 1-Test 3.

Code S2. Code for the selected Hui-Walter model including the covariance between Test 1-Test 3.

Code S3. Code for the selected Hui-Walter model including the covariance between Test 2-Test 3.

Table S3. Cross-tabulated test results by species and population.

Fig S2. Diagnostic plots for Model 1: performance of the tests.

Fig S3. Diagnostic plots for Model 1: prevalence across populations.

Fig S4. Plots of the sensitivity analysis for Models 1-3.

Fig S1. Results of the finite mixture models of the cutoff threshold for the MPB70 and IS6110 real-time PCR in isolates. The x-axis is log(cycle threshold), distribution of the positives in red and negatives in green.

MPB70

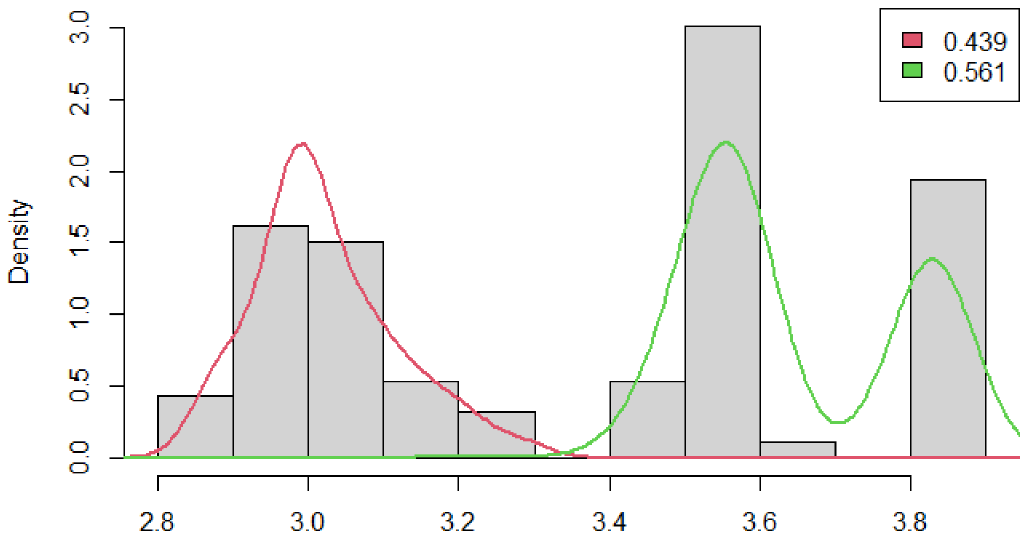

IS6110

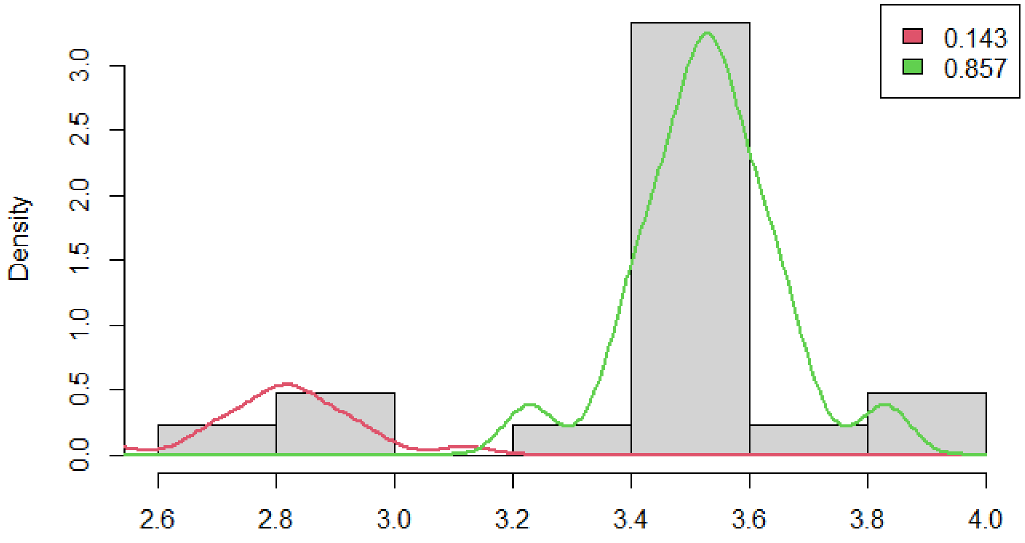

The cutoff threshold for positivity of the MPB70 and IS6110 real-time PCR protocols applied to bacterial suspensions was set using non-parametric finite mixture models as previously described (Pacheco et al., 2022), implemented using the package 'mixtools' (Benaglia et al., 2009). Finite mixture models allow to characterize the distributions of quantitative test results for the seropositive and seronegative subgroups within bimodal datasets (Benaglia et al., 2009). They are tools to estimate the probability of any given sample being positive or negative to a serological test, under a probabilistic diagnosis approach, without requiring reference samples of known infection status (Parker et al., 1990; Meyer et al., 2018; Lorenzi et al., 2019). The cutoff threshold in bacterial suspensions was set at Ct=28.5 for MPB70 and Ct=22.2 for IS6110 (Fig. S1).

References

T. Benaglia, D. Chauveau, D.R. Hunter, D.S. Young, mixtools: An R package for analyzing finite mixture models. J. Stat. Softw. 32 (2009) 1–29. https://doi.org/10.18637/jss.v032.i06

M. Lorenzi, M. Filippone, G.B. Frisoni, D.C. Alexander, S. Ourselin, Alzheimer's Disease Neuroimaging Initiative, Probabilistic disease progression modeling to characterize diagnostic uncertainty: application to staging and prediction in Alzheimer's disease. NeuroImage 190 (2019) 56-68. https://doi.org/10.1016/j.neuroimage.2017.08.059

A. Meyer, K. Bond, S. Van Winden, M. Green, J. Guitian, A probabilistic approach to the interpretation of milk antibody results for diagnosis of Johne's disease in dairy cattle. Prev. Vet. Med. 150 (2018) 30-37. https://doi.org/10.1016/j.prevetmed.2017.11.016

H. Pacheco, A.M. Lopes, J. Bárcena, E. Blanco, J. Abrantes, P. Esteves, R. Choquet, P.C. Alves, N. Santos, Multi-event capture–recapture models estimate the diagnostic performance of serological tests for myxoma and rabbit haemorrhagic disease viruses in the absence of reference samples. Transbound. Emerg. Dis. 69(5) (2022) e3024-e3035. https://doi.org/10.1111/tbed.14657

R.A. Parker, D.D. Erdman, L.J. Anderson, Use of mixture models in determining laboratory criterion for identification of seropositive individuals: application to parvovirus B19 serology. J. Virol. Meth. 27(2) (1990) 135-144. https://doi.org/10.1016/0166-0934(90)90130-8

Table S1. Beta distributions for the sensitivity and specificity of the diagnostic tests and their bibliographical support. Parameters of the beta distributions used as diffuse priors in Model 1 and Model 3.

Model	Host species	Diagnostic test	Sensitivity			Specificity		
			Beta distribution (α, β)	Median (CI_{95}) (%)	References	Beta distribution (α, β)	Median (CI_{95}) (%)	References
Model 1	Wild boar	Gross pathology	4.071056 1.856126	70.9 (30.1-95.6)	Santos et al., 2022	9.527001 1.477224	88.8 (62.2-99.0)	Santos et al., 2022
		Real-time PCR	4.981778 1.507261	79.6 (40.6-97.9)	Lorente-Leal et al., 2021	77.47122 6.141608	93.0 (86.2-97.2)	Lorente-Leal et al., 2021
		Bacteriological culture	3.101771 2.315743	58.2 (18.5-91.0)	Santos et al., 2022	34.45668 2.394028	94.3 (83.7-98.9)	Lorente-Leal et al., 2019
	Cervids	Gross pathology	7.81764 2.758008	75.5 (45.2-94.2)	Santos et al., 2022	13.24651 1.935955	88.9 (67.1-98.4)	Santos et al., 2022
		Real-time PCR	4.981778 1.507261	79.6 (40.6-97.9)	Lorente-Leal et al., 2021	77.47122 6.141608	93.0 (86.2-97.2)	Lorente-Leal et al., 2021
		Bacteriological culture	2.506658 2.073236	55.5 (14.2-91.5)	Santos et al., 2022	34.45668 2.394028	94.3 (83.7-98.9)	Lorente-Leal et al., 2019
Model 3	Wild boar	Gross pathology	18.67196 3.758051	90.1 (65.7-95.3)	Model 1	237.1672 5.084275	98.3 (95.8-99.3)	Model 1
		P22 indirect ELISA	26.66585 5.852403	84.1 (67.3-93.0)	Thomas et al., 2019a	96.33597 2.550178	98.4 (93.5-99.6)	Thomas et al., 2019a
		Bacteriological culture	16.53371 2.314131	87.2 (70.1-98.0)	Model 1	312.5549 3.51254	99.5 (97.5-99.7)	Model 1
	Cervids	Gross pathology	27.51271 7.92072	71.6 (62.8-89.6)	Model 1	75.38667 3.538532	96.3 (90.0-98.9)	Model 1
		P22 indirect ELISA	9.22462 4.508076	70.1 (41.3-88.3)	Thomas et al., 2019b	62.13694 1.617545	99.0 (92.5-99.8)	Thomas et al., 2019b
		Bacteriological culture	32.28187 14.15261	64.1 (55.7-81.7)	Model 1	312.5549 3.51254	99.2 (97.5-99.7)	Model 1

Table S2. Comparison of models including all combinations of pairwise covariances between diagnostic tests. Model 1 with diffuse priors extracted from the bibliography. Model 3 with diffuse priors extracted from the bibliography and from the posterior distributions of Model 1. Pairwise covariances selected for inference highlighted in bold.

**Pairwise covariances between tests included in the model**	**Model 1**			**Model 3**		
	**Deviance**	**Penalized deviance (DIC)**	**ΔDIC**	**Deviance**	**Penalized deviance (DIC)**	**ΔDIC**
No covariance between tests	125.6	144.1	0	147.7	162.8	9.0
T1-T3	**125.8**	**145.4**	**1.3**	140.2	157.1	3.3
T2-T3	126.4	146.0	1.9	**138.4**	**153.8**	**0**
T1-T2	127.0	146.7	2.6	144.5	160.4	6.6
T1-T3 + T2-T3	126.2	147.1	3.0	139.2	157.5	3.7
T1-T2 + T2-T3	127.1	148.0	3.9	138.4	155.7	1.9
T1-T2 + T1-T3	127.4	148.7	4.6	142.6	162.1	8.3
T1-T2 + T1-T3 + T2-T3 (full covariance)	127.3	149.5	5.4	143.2	165.5	11.7

Code S1. Code for the selected Hui-Walter model including the covariance between Test 1-Test 3. Code for Model 1.

```
Model1 <- c("model{
 pop_1 ~ dmulti(prob1, N_1)
 pop_2 ~ dmulti(prob2, N_2)
 pop_3 ~ dmulti(prob3, N_3)
 pop_4 ~ dmulti(prob4, N_4)
 pop_5 ~ dmulti(prob5, N_5)
 pop_6 ~ dmulti(prob6, N_6)
 pop_7 ~ dmulti(prob7, N_7)
 pop_8 ~ dmulti(prob8, N_8)
 pop_9 ~ dmulti(prob9, N_9)
 pop_10 ~ dmulti(prob10, N_10)
 pop_11 ~ dmulti(prob11, N_11)
 pop_12 ~ dmulti(prob12, N_12)
 pop_13 ~ dmulti(prob13, N_13)

 ## Wild boar
 # pop1 – site A
 prob1[2,2,2] <- prev[1]*(se[1]*se[2]*se[3]+cse13wb)+(1-prev[1])*((1-sp[1])*(1-sp[2])*(1-sp[3])+csp13wb)
 prob1[2,2,1] <- prev[1]*(se[1]*se[2]*(1-se[3])-cse13wb)+(1-prev[1])*((1-sp[1])*(1-sp[2])*sp[3]-csp13wb)
 prob1[2,1,2] <- prev[1]*(se[1]*(1-se[2])*se[3]+cse13wb)+(1-prev[1])*((1-sp[1])*sp[2]*(1-sp[3])+csp13wb)
 prob1[2,1,1] <- prev[1]*(se[1]*(1-se[2])*(1-se[3])-cse13wb)+(1-prev[1])*((1-sp[1])*sp[2]*sp[3]-csp13wb)
 prob1[1,2,2] <- prev[1]*((1-se[1])*se[2]*se[3]-cse13wb)+(1-prev[1])*(sp[1]*(1-sp[2])*(1-sp[3])-csp13wb)
 prob1[1,2,1] <- prev[1]*((1-se[1])*se[2]*(1-se[3])+cse13wb)+(1-prev[1])*(sp[1]*(1-sp[2])*sp[3]+csp13wb)
 prob1[1,1,2] <- prev[1]*((1-se[1])*(1-se[2])*se[3]-cse13wb)+(1-prev[1])*(sp[1]*sp[2]*(1-sp[3])-csp13wb)
 prob1[1,1,1] <- prev[1]*((1-se[1])*(1-se[2])*(1-se[3])+cse13wb)+(1-prev[1])*(sp[1]*sp[2]*sp[3]+csp13wb)

 # pop2 – site B
 prob2[2,2,2] <- prev[2]*(se[1]*se[2]*se[3]+cse13wb)+(1-prev[2])*((1-sp[1])*(1-sp[2])*(1-sp[3])+csp13wb)
 prob2[2,2,1] <- prev[2]*(se[1]*se[2]*(1-se[3])-cse13wb)+(1-prev[2])*((1-sp[1])*(1-sp[2])*sp[3]-csp13wb)
 prob2[2,1,2] <- prev[2]*(se[1]*(1-se[2])*se[3]+cse13wb)+(1-prev[2])*((1-sp[1])*sp[2]*(1-sp[3])+csp13wb)
 prob2[2,1,1] <- prev[2]*(se[1]*(1-se[2])*(1-se[3])-cse13wb)+(1-prev[2])*((1-sp[1])*sp[2]*sp[3]-csp13wb)
 prob2[1,2,2] <- prev[2]*((1-se[1])*se[2]*se[3]-cse13wb)+(1-prev[2])*(sp[1]*(1-sp[2])*(1-sp[3])-csp13wb)
 prob2[1,2,1] <- prev[2]*((1-se[1])*se[2]*(1-se[3])+cse13wb)+(1-prev[2])*(sp[1]*(1-sp[2])*sp[3]+csp13wb)
 prob2[1,1,2] <- prev[2]*((1-se[1])*(1-se[2])*se[3]-cse13wb)+(1-prev[2])*(sp[1]*sp[2]*(1-sp[3])-csp13wb)
 prob2[1,1,1] <- prev[2]*((1-se[1])*(1-se[2])*(1-se[3])+cse13wb)+(1-prev[2])*(sp[1]*sp[2]*sp[3]+csp13wb)

 # pop3 – site C
 prob3[2,2,2] <- prev[3]*(se[1]*se[2]*se[3]+cse13wb)+(1-prev[3])*((1-sp[1])*(1-sp[2])*(1-sp[3])+csp13wb)
 prob3[2,2,1] <- prev[3]*(se[1]*se[2]*(1-se[3])-cse13wb)+(1-prev[3])*((1-sp[1])*(1-sp[2])*sp[3]-csp13wb)
 prob3[2,1,2] <- prev[3]*(se[1]*(1-se[2])*se[3]+cse13wb)+(1-prev[3])*((1-sp[1])*sp[2]*(1-sp[3])+csp13wb)
 prob3[2,1,1] <- prev[3]*(se[1]*(1-se[2])*(1-se[3])-cse13wb)+(1-prev[3])*((1-sp[1])*sp[2]*sp[3]-csp13wb)
 prob3[1,2,2] <- prev[3]*((1-se[1])*se[2]*se[3]-cse13wb)+(1-prev[3])*(sp[1]*(1-sp[2])*(1-sp[3])-csp13wb)
 prob3[1,2,1] <- prev[3]*((1-se[1])*se[2]*(1-se[3])+cse13wb)+(1-prev[3])*(sp[1]*(1-sp[2])*sp[3]+csp13wb)
 prob3[1,1,2] <- prev[3]*((1-se[1])*(1-se[2])*se[3]-cse13wb)+(1-prev[3])*(sp[1]*sp[2]*(1-sp[3])-csp13wb)
 prob3[1,1,1] <- prev[3]*((1-se[1])*(1-se[2])*(1-se[3])+cse13wb)+(1-prev[3])*(sp[1]*sp[2]*sp[3]+csp13wb)

 # pop4 – site D
 prob4[2,2,2] <- prev[4]*(se[1]*se[2]*se[3]+cse13wb)+(1-prev[4])*((1-sp[1])*(1-sp[2])*(1-sp[3])+csp13wb)
 prob4[2,2,1] <- prev[4]*(se[1]*se[2]*(1-se[3])-cse13wb)+(1-prev[4])*((1-sp[1])*(1-sp[2])*sp[3]-csp13wb)
 prob4[2,1,2] <- prev[4]*(se[1]*(1-se[2])*se[3]+cse13wb)+(1-prev[4])*((1-sp[1])*sp[2]*(1-sp[3])+csp13wb)
 prob4[2,1,1] <- prev[4]*(se[1]*(1-se[2])*(1-se[3])-cse13wb)+(1-prev[4])*((1-sp[1])*sp[2]*sp[3]-csp13wb)
 prob4[1,2,2] <- prev[4]*((1-se[1])*se[2]*se[3]-cse13wb)+(1-prev[4])*(sp[1]*(1-sp[2])*(1-sp[3])-csp13wb)
 prob4[1,2,1] <- prev[4]*((1-se[1])*se[2]*(1-se[3])+cse13wb)+(1-prev[4])*(sp[1]*(1-sp[2])*sp[3]+csp13wb)
```

```
prob4[1,1,2] <- prev[4]*((1-se[1])*(1-se[2])*se[3]-cse13wb)+(1-prev[4])*(sp[1]*sp[2]*(1-sp[3])-csp13wb)
prob4[1,1,1] <- prev[4]*((1-se[1])*(1-se[2])*(1-se[3])+cse13wb)+(1-prev[4])*(sp[1]*sp[2]*sp[3]+csp13wb)

# pop5 - site E
prob5[2,2,2] <- prev[5]*(se[1]*se[2]*se[3]+cse13wb)+(1-prev[5])*((1-sp[1])*(1-sp[2])*(1-sp[3])+csp13wb)
prob5[2,2,1] <- prev[5]*(se[1]*se[2]*(1-se[3])-cse13wb)+(1-prev[5])*((1-sp[1])*(1-sp[2])*sp[3]-csp13wb)
prob5[2,1,2] <- prev[5]*(se[1]*(1-se[2])*se[3]+cse13wb)+(1-prev[5])*((1-sp[1])*sp[2]*(1-sp[3])+csp13wb)
prob5[2,1,1] <- prev[5]*(se[1]*(1-se[2])*(1-se[3])-cse13wb)+(1-prev[5])*((1-sp[1])*sp[2]*sp[3]-csp13wb)
prob5[1,2,2] <- prev[5]*((1-se[1])*se[2]*se[3]-cse13wb)+(1-prev[5])*(sp[1]*(1-sp[2])*(1-sp[3])-csp13wb)
prob5[1,2,1] <- prev[5]*((1-se[1])*se[2]*(1-se[3])+cse13wb)+(1-prev[5])*(sp[1]*(1-sp[2])*sp[3]+csp13wb)
prob5[1,1,2] <- prev[5]*((1-se[1])*(1-se[2])*se[3]-cse13wb)+(1-prev[5])*(sp[1]*sp[2]*(1-sp[3])-csp13wb)
prob5[1,1,1] <- prev[5]*((1-se[1])*(1-se[2])*(1-se[3])+cse13wb)+(1-prev[5])*(sp[1]*sp[2]*sp[3]+csp13wb)

# pop6 – site F
prob6[2,2,2] <- prev[6]*(se[1]*se[2]*se[3]+cse13wb)+(1-prev[6])*((1-sp[1])*(1-sp[2])*(1-sp[3])+csp13wb)
prob6[2,2,1] <- prev[6]*(se[1]*se[2]*(1-se[3])-cse13wb)+(1-prev[6])*((1-sp[1])*(1-sp[2])*sp[3]-csp13wb)
prob6[2,1,2] <- prev[6]*(se[1]*(1-se[2])*se[3]+cse13wb)+(1-prev[6])*((1-sp[1])*sp[2]*(1-sp[3])+csp13wb)
prob6[2,1,1] <- prev[6]*(se[1]*(1-se[2])*(1-se[3])-cse13wb)+(1-prev[6])*((1-sp[1])*sp[2]*sp[3]-csp13wb)
prob6[1,2,2] <- prev[6]*((1-se[1])*se[2]*se[3]-cse13wb)+(1-prev[6])*(sp[1]*(1-sp[2])*(1-sp[3])-csp13wb)
prob6[1,2,1] <- prev[6]*((1-se[1])*se[2]*(1-se[3])+cse13wb)+(1-prev[6])*(sp[1]*(1-sp[2])*sp[3]+csp13wb)
prob6[1,1,2] <- prev[6]*((1-se[1])*(1-se[2])*se[3]-cse13wb)+(1-prev[6])*(sp[1]*sp[2]*(1-sp[3])-csp13wb)
prob6[1,1,1] <- prev[6]*((1-se[1])*(1-se[2])*(1-se[3])+cse13wb)+(1-prev[6])*(sp[1]*sp[2]*sp[3]+csp13wb)

## Red deer
# pop7 – site A
prob7[2,2,2] <- prev[7]*(se2[1]*se2[2]*se2[3]+cse13rd)+(1-prev[7])*((1-sp2[1])*(1-sp2[2])*(1-sp2[3])+csp13rd)
prob7[2,2,1] <- prev[7]*(se2[1]*se2[2]*(1-se2[3])-cse13rd)+(1-prev[7])*((1-sp2[1])*(1-sp2[2])*sp2[3]-csp13rd)
prob7[2,1,2] <- prev[7]*(se2[1]*(1-se2[2])*se2[3]+cse13rd)+(1-prev[7])*((1-sp2[1])*sp2[2]*(1-sp2[3])+csp13rd)
prob7[2,1,1] <- prev[7]*(se2[1]*(1-se2[2])*(1-se2[3])-cse13rd)+(1-prev[7])*((1-sp2[1])*sp2[2]*sp2[3]-csp13rd)
prob7[1,2,2] <- prev[7]*((1-se2[1])*se2[2]*se2[3]-cse13rd)+(1-prev[7])*(sp2[1]*(1-sp2[2])*(1-sp2[3])-csp13rd)
prob7[1,2,1] <- prev[7]*((1-se2[1])*se2[2]*(1-se2[3])+cse13rd)+(1-prev[7])*(sp2[1]*(1-sp2[2])*sp2[3]+csp13rd)
prob7[1,1,2] <- prev[7]*((1-se2[1])*(1-se2[2])*se2[3]-cse13rd)+(1-prev[7])*(sp2[1]*sp2[2]*(1-sp2[3])-csp13rd)
prob7[1,1,1] <- prev[7]*((1-se2[1])*(1-se2[2])*(1-se2[3])+cse13rd)+(1-prev[7])*(sp2[1]*sp2[2]*sp2[3]+csp13rd)

# pop8 – site C
prob8[2,2,2] <- prev[8]*(se2[1]*se2[2]*se2[3]+cse13rd)+(1-prev[8])*((1-sp2[1])*(1-sp2[2])*(1-sp2[3])+csp13rd)
prob8[2,2,1] <- prev[8]*(se2[1]*se2[2]*(1-se2[3])-cse13rd)+(1-prev[8])*((1-sp2[1])*(1-sp2[2])*sp2[3]-csp13rd)
prob8[2,1,2] <- prev[8]*(se2[1]*(1-se2[2])*se2[3]+cse13rd)+(1-prev[8])*((1-sp2[1])*sp2[2]*(1-sp2[3])+csp13rd)
prob8[2,1,1] <- prev[8]*(se2[1]*(1-se2[2])*(1-se2[3])-cse13rd)+(1-prev[8])*((1-sp2[1])*sp2[2]*sp2[3]-csp13rd)
prob8[1,2,2] <- prev[8]*((1-se2[1])*se2[2]*se2[3]-cse13rd)+(1-prev[8])*(sp2[1]*(1-sp2[2])*(1-sp2[3])-csp13rd)
prob8[1,2,1] <- prev[8]*((1-se2[1])*se2[2]*(1-se2[3])+cse13rd)+(1-prev[8])*(sp2[1]*(1-sp2[2])*sp2[3]+csp13rd)
prob8[1,1,2] <- prev[8]*((1-se2[1])*(1-se2[2])*se2[3]-cse13rd)+(1-prev[8])*(sp2[1]*sp2[2]*(1-sp2[3])-csp13rd)
prob8[1,1,1] <- prev[8]*((1-se2[1])*(1-se2[2])*(1-se2[3])+cse13rd)+(1-prev[8])*(sp2[1]*sp2[2]*sp2[3]+csp13rd)

# pop9 – site D
prob9[2,2,2] <- prev[9]*(se2[1]*se2[2]*se2[3]+cse13rd)+(1-prev[9])*((1-sp2[1])*(1-sp2[2])*(1-sp2[3])+csp13rd)
prob9[2,2,1] <- prev[9]*(se2[1]*se2[2]*(1-se2[3])-cse13rd)+(1-prev[9])*((1-sp2[1])*(1-sp2[2])*sp2[3]-csp13rd)
prob9[2,1,2] <- prev[9]*(se2[1]*(1-se2[2])*se2[3]+cse13rd)+(1-prev[9])*((1-sp2[1])*sp2[2]*(1-sp2[3])+csp13rd)
prob9[2,1,1] <- prev[9]*(se2[1]*(1-se2[2])*(1-se2[3])-cse13rd)+(1-prev[9])*((1-sp2[1])*sp2[2]*sp2[3]-csp13rd)
prob9[1,2,2] <- prev[9]*((1-se2[1])*se2[2]*se2[3]-cse13rd)+(1-prev[9])*(sp2[1]*(1-sp2[2])*(1-sp2[3])-csp13rd)
prob9[1,2,1] <- prev[9]*((1-se2[1])*se2[2]*(1-se2[3])+cse13rd)+(1-prev[9])*(sp2[1]*(1-sp2[2])*sp2[3]+csp13rd)
prob9[1,1,2] <- prev[9]*((1-se2[1])*(1-se2[2])*se2[3]-cse13rd)+(1-prev[9])*(sp2[1]*sp2[2]*(1-sp2[3])-csp13rd)
prob9[1,1,1] <- prev[9]*((1-se2[1])*(1-se2[2])*(1-se2[3])+cse13rd)+(1-prev[9])*(sp2[1]*sp2[2]*sp2[3]+csp13rd)

# pop10 – site E
prob10[2,2,2] <- prev[10]*(se2[1]*se2[2]*se2[3]+cse13rd)+(1-prev[10])*((1-sp2[1])*(1-sp2[2])*(1-sp2[3])+csp13rd)
prob10[2,2,1] <- prev[10]*(se2[1]*se2[2]*(1-se2[3])-cse13rd)+(1-prev[10])*((1-sp2[1])*(1-sp2[2])*sp2[3]-csp13rd)
prob10[2,1,2] <- prev[10]*(se2[1]*(1-se2[2])*se2[3]+cse13rd)+(1-prev[10])*((1-sp2[1])*sp2[2]*(1-sp2[3])+csp13rd)
```

```
prob10[2,1,1] <- prev[10]*(se2[1]*(1-se2[2])*(1-se2[3])-cse13rd)+(1-prev[10])*((1-sp2[1])*sp2[2]*sp2[3]-csp13rd)
prob10[1,2,2] <- prev[10]*((1-se2[1])*se2[2]*se2[3]-cse13rd)+(1-prev[10])*(sp2[1]*(1-sp2[2])*(1-sp2[3])-csp13rd)
prob10[1,2,1] <- prev[10]*((1-se2[1])*se2[2]*(1-se2[3])+cse13rd)+(1-prev[10])*(sp2[1]*(1-sp2[2])*sp2[3]+csp13rd)
prob10[1,1,2] <- prev[10]*((1-se2[1])*(1-se2[2])*se2[3]-cse13rd)+(1-prev[10])*(sp2[1]*sp2[2]*(1-sp2[3])-csp13rd)
prob10[1,1,1] <- prev[10]*((1-se2[1])*(1-se2[2])*(1-se2[3])+cse13rd)+(1-prev[10])*(sp2[1]*sp2[2]*sp2[3]+csp13rd)

# pop11 – site F
prob11[2,2,2] <- prev[11]*(se2[1]*se2[2]*se2[3]+cse13rd)+(1-prev[11])*((1-sp2[1])*(1-sp2[2])*(1-sp2[3])+csp13rd)
prob11[2,2,1] <- prev[11]*(se2[1]*se2[2]*(1-se2[3])-cse13rd)+(1-prev[11])*((1-sp2[1])*(1-sp2[2])*sp2[3]-csp13rd)
prob11[2,1,2] <- prev[11]*(se2[1]*(1-se2[2])*se2[3]+cse13rd)+(1-prev[11])*((1-sp2[1])*sp2[2]*(1-sp2[3])+csp13rd)
prob11[2,1,1] <- prev[11]*(se2[1]*(1-se2[2])*(1-se2[3])-cse13rd)+(1-prev[11])*((1-sp2[1])*sp2[2]*sp2[3]-csp13rd)
prob11[1,2,2] <- prev[11]*((1-se2[1])*se2[2]*se2[3]-cse13rd)+(1-prev[11])*(sp2[1]*(1-sp2[2])*(1-sp2[3])-csp13rd)
prob11[1,2,1] <- prev[11]*((1-se2[1])*se2[2]*(1-se2[3])+cse13rd)+(1-prev[11])*(sp2[1]*(1-sp2[2])*sp2[3]+csp13rd)
prob11[1,1,2] <- prev[11]*((1-se2[1])*(1-se2[2])*se2[3]-cse13rd)+(1-prev[11])*(sp2[1]*sp2[2]*(1-sp2[3])-csp13rd)
prob11[1,1,1] <- prev[11]*((1-se2[1])*(1-se2[2])*(1-se2[3])+cse13rd)+(1-prev[11])*(sp2[1]*sp2[2]*sp2[3]+csp13rd)

## Fallow deer
# pop12 – site C
prob12[2,2,2] <- prev[12]*(se2[1]*se2[2]*se2[3]+cse13rd)+(1-prev[12])*((1-sp2[1])*(1-sp2[2])*(1-sp2[3])+csp13rd)
prob12[2,2,1] <- prev[12]*(se2[1]*se2[2]*(1-se2[3])-cse13rd)+(1-prev[12])*((1-sp2[1])*(1-sp2[2])*sp2[3]-csp13rd)
prob12[2,1,2] <- prev[12]*(se2[1]*(1-se2[2])*se2[3]+cse13rd)+(1-prev[12])*((1-sp2[1])*sp2[2]*(1-sp2[3])+csp13rd)
prob12[2,1,1] <- prev[12]*(se2[1]*(1-se2[2])*(1-se2[3])-cse13rd)+(1-prev[12])*((1-sp2[1])*sp2[2]*sp2[3]-csp13rd)
prob12[1,2,2] <- prev[12]*((1-se2[1])*se2[2]*se2[3]-cse13rd)+(1-prev[12])*(sp2[1]*(1-sp2[2])*(1-sp2[3])-csp13rd)
prob12[1,2,1] <- prev[12]*((1-se2[1])*se2[2]*(1-se2[3])+cse13rd)+(1-prev[12])*(sp2[1]*(1-sp2[2])*sp2[3]+csp13rd)
prob12[1,1,2] <- prev[12]*((1-se2[1])*(1-se2[2])*se2[3]-cse13rd)+(1-prev[12])*(sp2[1]*sp2[2]*(1-sp2[3])-csp13rd)
prob12[1,1,1] <- prev[12]*((1-se2[1])*(1-se2[2])*(1-se2[3])+cse13rd)+(1-prev[12])*(sp2[1]*sp2[2]*sp2[3]+csp13rd)

# pop13 – site D
prob13[2,2,2] <- prev[13]*(se2[1]*se2[2]*se2[3]+cse13rd)+(1-prev[13])*((1-sp2[1])*(1-sp2[2])*(1-sp2[3])+csp13rd)
prob13[2,2,1] <- prev[13]*(se2[1]*se2[2]*(1-se2[3])-cse13rd)+(1-prev[13])*((1-sp2[1])*(1-sp2[2])*sp2[3]-csp13rd)
prob13[2,1,2] <- prev[13]*(se2[1]*(1-se2[2])*se2[3]+cse13rd)+(1-prev[13])*((1-sp2[1])*sp2[2]*(1-sp2[3])+csp13rd)
prob13[2,1,1] <- prev[13]*(se2[1]*(1-se2[2])*(1-se2[3])-cse13rd)+(1-prev[13])*((1-sp2[1])*sp2[2]*sp2[3]-csp13rd)
prob13[1,2,2] <- prev[13]*((1-se2[1])*se2[2]*se2[3]-cse13rd)+(1-prev[13])*(sp2[1]*(1-sp2[2])*(1-sp2[3])-csp13rd)
prob13[1,2,1] <- prev[13]*((1-se2[1])*se2[2]*(1-se2[3])+cse13rd)+(1-prev[13])*(sp2[1]*(1-sp2[2])*sp2[3]+csp13rd)
prob13[1,1,2] <- prev[13]*((1-se2[1])*(1-se2[2])*se2[3]-cse13rd)+(1-prev[13])*(sp2[1]*sp2[2]*(1-sp2[3])-csp13rd)
prob13[1,1,1] <- prev[13]*((1-se2[1])*(1-se2[2])*(1-se2[3])+cse13rd)+(1-prev[13])*(sp2[1]*sp2[2]*sp2[3]+csp13rd)

## Priors
# Prevalence
prev[1] ~ dbeta(0.5, 0.5)
prev[2] ~ dbeta(0.5, 0.5)
prev[3] ~ dbeta(0.5, 0.5)
prev[4] ~ dbeta(0.5, 0.5)
prev[5] ~ dbeta(0.5, 0.5)
prev[6] ~ dbeta(0.5, 0.5)
prev[7] ~ dbeta(0.5, 0.5)
prev[8] ~ dbeta(0.5, 0.5)
prev[9] ~ dbeta(0.5, 0.5)
prev[10] ~ dbeta(0.5, 0.5)
prev[11] ~ dbeta(0.5, 0.5)
prev[12] ~ dbeta(0.5, 0.5)
prev[13] ~ dbeta(0.5, 0.5)

# Se and Sp
se[1] ~ dbeta(4.071056, 1.856126)T(1-sp[1], )          # Se gross pathology Wild boar
sp[1] ~ dbeta(9.527001, 1.477224)                      # Sp gross pathology Wild boar
se[2] ~ dbeta(4.981778, 1.507261)T(1-sp[2], )          # Se PCR IS6110 Wild boar
sp[2] ~ dbeta(77.47122, 6.141608)                      # Sp PCR IS6110 Wild boar
```

```
  se[3] ~ dbeta(3.101771, 2.315743)T(1-sp[3], )                  # Se bacteriology Wild boar
  sp[3] ~ dbeta(34.45668, 2.394028)                              # Sp bacteriology Wild boar
  se2[1] ~ dbeta(7.81764, 2.758008)T(1-sp2[1], )                 # Se gross pathology Cervids
  sp2[1] ~ dbeta(13.24651, 1.935955)                             # Sp gross pathology Cervids
  se2[2] ~ dbeta(4.981778, 1.507261)T(1-sp2[2], )                # Se PCR IS6110 Cervids
  sp2[2] ~ dbeta(77.47122, 6.141608)                             # Sp PCR IS6110 Cervids
  se2[3] ~ dbeta(2.506658, 2.073236)T(1-sp2[3], )                # Se bacteriology Cervids
  sp2[3] ~ dbeta(34.45668, 2.394028)                             # Sp bacteriology Cervids

  ## Covariances
  cse13wb ~ dunif((se[1]-1)*(1-se[3]), min(se[1], se[3]) - se[1]*se[3])              # covariance between Se T1-T3 Wild boar
  csp13wb ~ dunif((sp[1]-1)*(1-sp[3]), min(sp[1], sp[3]) - sp[1]*sp[3])              # covariance between Sp T1-T3 Wild boar
  cse13rd ~ dunif((se2[1]-1)*(1-se2[3]), min(se2[1], se2[3]) - se2[1]*se2[3])        # covariance between Se T1-T3 Cervids
  csp13rd ~ dunif((sp2[1]-1)*(1-sp2[3]), min(sp2[1], sp2[3]) - sp2[1]*sp2[3])        # covariance between Sp T1-T3 Cervids

  ## Correlations
  cor13pwb <- cse13wb / sqrt(se[1]*(1-se[1])*se[3]*(1-se[3]))                        # correlation between Se T1-T3 Wild boar
  cor13nwb <- csp13wb / sqrt(sp[1]*(1-sp[1])*sp[3]*(1-sp[3]))                        # correlation between Sp T1-T3 Wild boar
  cor13prd <- cse13rd / sqrt(se2[1]*(1-se2[1])*se2[3]*(1-se2[3]))                    # correlation between Se T1-T3 Cervids
  cor13nrd <- csp13rd / sqrt(sp2[1]*(1-sp2[1])*sp2[3]*(1-sp2[3]))                    # correlation between Sp T1-T3 Cervids

  ## Derived parameters
  # Parallel interpretation T1-T3 cervids
  se_par<-1-((1-se2[1])*(1-se2[3]))
  sp_par<-sp2[1]*sp2[3]

  #data# pop_1, pop_2, pop_3, pop_4, pop_5, pop_6, pop_7, pop_8, pop_9, pop_10, pop_11, pop_12, pop_13, N_1, N_2, N_3, N_4, N_5, N_6,
N_7, N_8, N_9, N_10, N_11, N_12, N_13

  #monitor# prev, se, sp, se2, sp2, cse13wb, csp13wb, cor13pwb, cor13nwb, cse13rd, csp13rd, cor13prd, cor13nrd,  se_par, sp_par

  #inits# prev, se, sp, se2, sp2, cse13wb, csp13wb, cse13rd, csp13rd

}
")
```

Code S2. Code for the selected Hui-Walter model including the covariance between Test 1-Test 3. Code for Model 2.

```
model2 <- c("model{
 pop_1 ~ dmulti(prob1, N_1)
 pop_2 ~ dmulti(prob2, N_2)
 pop_3 ~ dmulti(prob3, N_3)
 pop_4 ~ dmulti(prob4, N_4)
 pop_5 ~ dmulti(prob5, N_5)
 pop_6 ~ dmulti(prob6, N_6)
 pop_7 ~ dmulti(prob7, N_7)
 pop_8 ~ dmulti(prob8, N_8)
 pop_9 ~ dmulti(prob9, N_9)
 pop_10 ~ dmulti(prob10, N_10)
 pop_11 ~ dmulti(prob11, N_11)
 pop_12 ~ dmulti(prob12, N_12)
 pop_13 ~ dmulti(prob13, N_13)

 # wild boar
 # pop1 – site A
 prob1[2,2,2] <- prev[1]*(se[1]*se[2]*se[3]+cse13wb)+(1-prev[1])*((1-sp[1])*(1-sp[2])*(1-sp[3])+csp13wb)
 prob1[2,2,1] <- prev[1]*(se[1]*se[2]*(1-se[3])-cse13wb)+(1-prev[1])*((1-sp[1])*(1-sp[2])*sp[3]-csp13wb)
 prob1[2,1,2] <- prev[1]*(se[1]*(1-se[2])*se[3]+cse13wb)+(1-prev[1])*((1-sp[1])*sp[2]*(1-sp[3])+csp13wb)
 prob1[2,1,1] <- prev[1]*(se[1]*(1-se[2])*(1-se[3])-cse13wb)+(1-prev[1])*((1-sp[1])*sp[2]*sp[3]-csp13wb)
 prob1[1,2,2] <- prev[1]*((1-se[1])*se[2]*se[3]-cse13wb)+(1-prev[1])*(sp[1]*(1-sp[2])*(1-sp[3])-csp13wb)
 prob1[1,2,1] <- prev[1]*((1-se[1])*se[2]*(1-se[3])+cse13wb)+(1-prev[1])*(sp[1]*(1-sp[2])*sp[3]+csp13wb)
 prob1[1,1,2] <- prev[1]*((1-se[1])*(1-se[2])*se[3]-cse13wb)+(1-prev[1])*(sp[1]*sp[2]*(1-sp[3])-csp13wb)
 prob1[1,1,1] <- prev[1]*((1-se[1])*(1-se[2])*(1-se[3])+cse13wb)+(1-prev[1])*(sp[1]*sp[2]*sp[3]+csp13wb)

 # pop2 – site B
 prob2[2,2,2] <- prev[2]*(se[1]*se[2]*se[3]+cse13wb)+(1-prev[2])*((1-sp[1])*(1-sp[2])*(1-sp[3])+csp13wb)
 prob2[2,2,1] <- prev[2]*(se[1]*se[2]*(1-se[3])-cse13wb)+(1-prev[2])*((1-sp[1])*(1-sp[2])*sp[3]-csp13wb)
 prob2[2,1,2] <- prev[2]*(se[1]*(1-se[2])*se[3]+cse13wb)+(1-prev[2])*((1-sp[1])*sp[2]*(1-sp[3])+csp13wb)
 prob2[2,1,1] <- prev[2]*(se[1]*(1-se[2])*(1-se[3])-cse13wb)+(1-prev[2])*((1-sp[1])*sp[2]*sp[3]-csp13wb)
 prob2[1,2,2] <- prev[2]*((1-se[1])*se[2]*se[3]-cse13wb)+(1-prev[2])*(sp[1]*(1-sp[2])*(1-sp[3])-csp13wb)
 prob2[1,2,1] <- prev[2]*((1-se[1])*se[2]*(1-se[3])+cse13wb)+(1-prev[2])*(sp[1]*(1-sp[2])*sp[3]+csp13wb)
 prob2[1,1,2] <- prev[2]*((1-se[1])*(1-se[2])*se[3]-cse13wb)+(1-prev[2])*(sp[1]*sp[2]*(1-sp[3])-csp13wb)
 prob2[1,1,1] <- prev[2]*((1-se[1])*(1-se[2])*(1-se[3])+cse13wb)+(1-prev[2])*(sp[1]*sp[2]*sp[3]+csp13wb)

 # pop3 – site C
 prob3[2,2,2] <- prev[3]*(se[1]*se[2]*se[3]+cse13wb)+(1-prev[3])*((1-sp[1])*(1-sp[2])*(1-sp[3])+csp13wb)
 prob3[2,2,1] <- prev[3]*(se[1]*se[2]*(1-se[3])-cse13wb)+(1-prev[3])*((1-sp[1])*(1-sp[2])*sp[3]-csp13wb)
 prob3[2,1,2] <- prev[3]*(se[1]*(1-se[2])*se[3]+cse13wb)+(1-prev[3])*((1-sp[1])*sp[2]*(1-sp[3])+csp13wb)
 prob3[2,1,1] <- prev[3]*(se[1]*(1-se[2])*(1-se[3])-cse13wb)+(1-prev[3])*((1-sp[1])*sp[2]*sp[3]-csp13wb)
 prob3[1,2,2] <- prev[3]*((1-se[1])*se[2]*se[3]-cse13wb)+(1-prev[3])*(sp[1]*(1-sp[2])*(1-sp[3])-csp13wb)
 prob3[1,2,1] <- prev[3]*((1-se[1])*se[2]*(1-se[3])+cse13wb)+(1-prev[3])*(sp[1]*(1-sp[2])*sp[3]+csp13wb)
 prob3[1,1,2] <- prev[3]*((1-se[1])*(1-se[2])*se[3]-cse13wb)+(1-prev[3])*(sp[1]*sp[2]*(1-sp[3])-csp13wb)
 prob3[1,1,1] <- prev[3]*((1-se[1])*(1-se[2])*(1-se[3])+cse13wb)+(1-prev[3])*(sp[1]*sp[2]*sp[3]+csp13wb)

 # pop4 – site D
 prob4[2,2,2] <- prev[4]*(se[1]*se[2]*se[3]+cse13wb)+(1-prev[4])*((1-sp[1])*(1-sp[2])*(1-sp[3])+csp13wb)
 prob4[2,2,1] <- prev[4]*(se[1]*se[2]*(1-se[3])-cse13wb)+(1-prev[4])*((1-sp[1])*(1-sp[2])*sp[3]-csp13wb)
 prob4[2,1,2] <- prev[4]*(se[1]*(1-se[2])*se[3]+cse13wb)+(1-prev[4])*((1-sp[1])*sp[2]*(1-sp[3])+csp13wb)
 prob4[2,1,1] <- prev[4]*(se[1]*(1-se[2])*(1-se[3])-cse13wb)+(1-prev[4])*((1-sp[1])*sp[2]*sp[3]-csp13wb)
 prob4[1,2,2] <- prev[4]*((1-se[1])*se[2]*se[3]-cse13wb)+(1-prev[4])*(sp[1]*(1-sp[2])*(1-sp[3])-csp13wb)
 prob4[1,2,1] <- prev[4]*((1-se[1])*se[2]*(1-se[3])+cse13wb)+(1-prev[4])*(sp[1]*(1-sp[2])*sp[3]+csp13wb)
 prob4[1,1,2] <- prev[4]*((1-se[1])*(1-se[2])*se[3]-cse13wb)+(1-prev[4])*(sp[1]*sp[2]*(1-sp[3])-csp13wb)
```

```
prob4[1,1,1] <- prev[4]*((1-se[1])*(1-se[2])*(1-se[3])+cse13wb)+(1-prev[4])*(sp[1]*sp[2]*sp[3]+csp13wb)

# pop5 – site E
prob5[2,2,2] <- prev[5]*(se[1]*se[2]*se[3]+cse13wb)+(1-prev[5])*((1-sp[1])*(1-sp[2])*(1-sp[3])+csp13wb)
prob5[2,2,1] <- prev[5]*(se[1]*se[2]*(1-se[3])-cse13wb)+(1-prev[5])*((1-sp[1])*(1-sp[2])*sp[3]-csp13wb)
prob5[2,1,2] <- prev[5]*(se[1]*(1-se[2])*se[3]+cse13wb)+(1-prev[5])*((1-sp[1])*sp[2]*(1-sp[3])+csp13wb)
prob5[2,1,1] <- prev[5]*(se[1]*(1-se[2])*(1-se[3])-cse13wb)+(1-prev[5])*((1-sp[1])*sp[2]*sp[3]-csp13wb)
prob5[1,2,2] <- prev[5]*((1-se[1])*se[2]*se[3]-cse13wb)+(1-prev[5])*(sp[1]*(1-sp[2])*(1-sp[3])-csp13wb)
prob5[1,2,1] <- prev[5]*((1-se[1])*se[2]*(1-se[3])+cse13wb)+(1-prev[5])*(sp[1]*(1-sp[2])*sp[3]+csp13wb)
prob5[1,1,2] <- prev[5]*((1-se[1])*(1-se[2])*se[3]-cse13wb)+(1-prev[5])*(sp[1]*sp[2]*(1-sp[3])-csp13wb)
prob5[1,1,1] <- prev[5]*((1-se[1])*(1-se[2])*(1-se[3])+cse13wb)+(1-prev[5])*(sp[1]*sp[2]*sp[3]+csp13wb)

# pop6 – site F
prob6[2,2,2] <- prev[6]*(seh[1]*seh[2]*seh[3]+cse13wb)+(1-prev[6])*((1-sp[1])*(1-sp[2])*(1-sp[3])+csp13wb)
prob6[2,2,1] <- prev[6]*(seh[1]*seh[2]*(1-seh[3])-cse13wb)+(1-prev[6])*((1-sp[1])*(1-sp[2])*sp[3]-csp13wb)
prob6[2,1,2] <- prev[6]*(seh[1]*(1-seh[2])*seh[3]+cse13wb)+(1-prev[6])*((1-sp[1])*sp[2]*(1-sp[3])+csp13wb)
prob6[2,1,1] <- prev[6]*(seh[1]*(1-seh[2])*(1-seh[3])-cse13wb)+(1-prev[6])*((1-sp[1])*sp[2]*sp[3]-csp13wb)
prob6[1,2,2] <- prev[6]*((1-seh[1])*seh[2]*seh[3]-cse13wb)+(1-prev[6])*(sp[1]*(1-sp[2])*(1-sp[3])-csp13wb)
prob6[1,2,1] <- prev[6]*((1-seh[1])*seh[2]*(1-seh[3])+cse13wb)+(1-prev[6])*(sp[1]*(1-sp[2])*sp[3]+csp13wb)
prob6[1,1,2] <- prev[6]*((1-seh[1])*(1-seh[2])*seh[3]-cse13wb)+(1-prev[6])*(sp[1]*sp[2]*(1-sp[3])-csp13wb)
prob6[1,1,1] <- prev[6]*((1-seh[1])*(1-seh[2])*(1-seh[3])+cse13wb)+(1-prev[6])*(sp[1]*sp[2]*sp[3]+csp13wb)

# red deer
# pop7 – site A
prob7[2,2,2] <- prev[7]*(se2[1]*se2[2]*se2[3]+cse13rd)+(1-prev[7])*((1-sp[1])*(1-sp[2])*(1-sp[3])+csp13rd)
prob7[2,2,1] <- prev[7]*(se2[1]*se2[2]*(1-se2[3])-cse13rd)+(1-prev[7])*((1-sp[1])*(1-sp[2])*sp[3]-csp13rd)
prob7[2,1,2] <- prev[7]*(se2[1]*(1-se2[2])*se2[3]+cse13rd)+(1-prev[7])*((1-sp[1])*sp[2]*(1-sp[3])+csp13rd)
prob7[2,1,1] <- prev[7]*(se2[1]*(1-se2[2])*(1-se2[3])-cse13rd)+(1-prev[7])*((1-sp[1])*sp[2]*sp[3]-csp13rd)
prob7[1,2,2] <- prev[7]*((1-se2[1])*se2[2]*se2[3]-cse13rd)+(1-prev[7])*(sp[1]*(1-sp[2])*(1-sp[3])-csp13rd)
prob7[1,2,1] <- prev[7]*((1-se2[1])*se2[2]*(1-se2[3])+cse13rd)+(1-prev[7])*(sp[1]*(1-sp[2])*sp[3]+csp13rd)
prob7[1,1,2] <- prev[7]*((1-se2[1])*(1-se2[2])*se2[3]-cse13rd)+(1-prev[7])*(sp[1]*sp[2]*(1-sp[3])-csp13rd)
prob7[1,1,1] <- prev[7]*((1-se2[1])*(1-se2[2])*(1-se2[3])+cse13rd)+(1-prev[7])*(sp[1]*sp[2]*sp[3]+csp13rd)

# pop8 – site C
prob8[2,2,2] <- prev[8]*(se2[1]*se2[2]*se2[3]+cse13rd)+(1-prev[8])*((1-sp[1])*(1-sp[2])*(1-sp[3])+csp13rd)
prob8[2,2,1] <- prev[8]*(se2[1]*se2[2]*(1-se2[3])-cse13rd)+(1-prev[8])*((1-sp[1])*(1-sp[2])*sp[3]-csp13rd)
prob8[2,1,2] <- prev[8]*(se2[1]*(1-se2[2])*se2[3]+cse13rd)+(1-prev[8])*((1-sp[1])*sp[2]*(1-sp[3])+csp13rd)
prob8[2,1,1] <- prev[8]*(se2[1]*(1-se2[2])*(1-se2[3])-cse13rd)+(1-prev[8])*((1-sp[1])*sp[2]*sp[3]-csp13rd)
prob8[1,2,2] <- prev[8]*((1-se2[1])*se2[2]*se2[3]-cse13rd)+(1-prev[8])*(sp[1]*(1-sp[2])*(1-sp[3])-csp13rd)
prob8[1,2,1] <- prev[8]*((1-se2[1])*se2[2]*(1-se2[3])+cse13rd)+(1-prev[8])*(sp[1]*(1-sp[2])*sp[3]+csp13rd)
prob8[1,1,2] <- prev[8]*((1-se2[1])*(1-se2[2])*se2[3]-cse13rd)+(1-prev[8])*(sp[1]*sp[2]*(1-sp[3])-csp13rd)
prob8[1,1,1] <- prev[8]*((1-se2[1])*(1-se2[2])*(1-se2[3])+cse13rd)+(1-prev[8])*(sp[1]*sp[2]*sp[3]+csp13rd)

# pop9 – site D
prob9[2,2,2] <- prev[9]*(se2h[1]*se2h[2]*se2h[3]+cse13rd)+(1-prev[9])*((1-sp[1])*(1-sp[2])*(1-sp[3])+csp13rd)
prob9[2,2,1] <- prev[9]*(se2h[1]*se2h[2]*(1-se2h[3])-cse13rd)+(1-prev[9])*((1-sp[1])*(1-sp[2])*sp[3]-csp13rd)
prob9[2,1,2] <- prev[9]*(se2h[1]*(1-se2h[2])*se2h[3]+cse13rd)+(1-prev[9])*((1-sp[1])*sp[2]*(1-sp[3])+csp13rd)
prob9[2,1,1] <- prev[9]*(se2h[1]*(1-se2h[2])*(1-se2h[3])-cse13rd)+(1-prev[9])*((1-sp[1])*sp[2]*sp[3]-csp13rd)
prob9[1,2,2] <- prev[9]*((1-se2h[1])*se2h[2]*se2h[3]-cse13rd)+(1-prev[9])*(sp[1]*(1-sp[2])*(1-sp[3])-csp13rd)
prob9[1,2,1] <- prev[9]*((1-se2h[1])*se2h[2]*(1-se2h[3])+cse13rd)+(1-prev[9])*(sp[1]*(1-sp[2])*sp[3]+csp13rd)
prob9[1,1,2] <- prev[9]*((1-se2h[1])*(1-se2h[2])*se2h[3]-cse13rd)+(1-prev[9])*(sp[1]*sp[2]*(1-sp[3])-csp13rd)
prob9[1,1,1] <- prev[9]*((1-se2h[1])*(1-se2h[2])*(1-se2h[3])+cse13rd)+(1-prev[9])*(sp[1]*sp[2]*sp[3]+csp13rd)

# pop10 – site E
prob10[2,2,2] <- prev[10]*(se2[1]*se2[2]*se2[3]+cse13rd)+(1-prev[10])*((1-sp[1])*(1-sp[2])*(1-sp[3])+csp13rd)
prob10[2,2,1] <- prev[10]*(se2[1]*se2[2]*(1-se2[3])-cse13rd)+(1-prev[10])*((1-sp[1])*(1-sp[2])*sp[3]-csp13rd)
prob10[2,1,2] <- prev[10]*(se2[1]*(1-se2[2])*se2[3]+cse13rd)+(1-prev[10])*((1-sp[1])*sp[2]*(1-sp[3])+csp13rd)
prob10[2,1,1] <- prev[10]*(se2[1]*(1-se2[2])*(1-se2[3])-cse13rd)+(1-prev[10])*((1-sp[1])*sp[2]*sp[3]-csp13rd)
```

```
prob10[1,2,2] <- prev[10]*((1-se2[1])*se2[2]*se2[3]-cse13rd)+(1-prev[10])*(sp[1]*(1-sp[2])*(1-sp[3])-csp13rd)
prob10[1,2,1] <- prev[10]*((1-se2[1])*se2[2]*(1-se2[3])+cse13rd)+(1-prev[10])*(sp[1]*(1-sp[2])*sp[3]+csp13rd)
prob10[1,1,2] <- prev[10]*((1-se2[1])*(1-se2[2])*se2[3]-cse13rd)+(1-prev[10])*(sp[1]*sp[2]*(1-sp[3])-csp13rd)
prob10[1,1,1] <- prev[10]*((1-se2[1])*(1-se2[2])*(1-se2[3])+cse13rd)+(1-prev[10])*(sp[1]*sp[2]*sp[3]+csp13rd)

# pop11 – site F
prob11[2,2,2] <- prev[11]*(se2h[1]*se2h[2]*se2h[3]+cse13rd)+(1-prev[11])*((1-sp[1])*(1-sp[2])*(1-sp[3])+csp13rd)
prob11[2,2,1] <- prev[11]*(se2h[1]*se2h[2]*(1-se2h[3])-cse13rd)+(1-prev[11])*((1-sp[1])*(1-sp[2])*sp[3]-csp13rd)
prob11[2,1,2] <- prev[11]*(se2h[1]*(1-se2h[2])*se2h[3]+cse13rd)+(1-prev[11])*((1-sp[1])*sp[2]*(1-sp[3])+csp13rd)
prob11[2,1,1] <- prev[11]*(se2h[1]*(1-se2h[2])*(1-se2h[3])-cse13rd)+(1-prev[11])*((1-sp[1])*sp[2]*sp[3]-csp13rd)
prob11[1,2,2] <- prev[11]*((1-se2h[1])*se2h[2]*se2h[3]-cse13rd)+(1-prev[11])*(sp[1]*(1-sp[2])*(1-sp[3])-csp13rd)
prob11[1,2,1] <- prev[11]*((1-se2h[1])*se2h[2]*(1-se2h[3])+cse13rd)+(1-prev[11])*(sp[1]*(1-sp[2])*sp[3]+csp13rd)
prob11[1,1,2] <- prev[11]*((1-se2h[1])*(1-se2h[2])*se2h[3]-cse13rd)+(1-prev[11])*(sp[1]*sp[2]*(1-sp[3])-csp13rd)
prob11[1,1,1] <- prev[11]*((1-se2h[1])*(1-se2h[2])*(1-se2h[3])+cse13rd)+(1-prev[11])*(sp[1]*sp[2]*sp[3]+csp13rd)

# fallow deer
# pop12 – site C
prob12[2,2,2] <- prev[12]*(se2h[1]*se2h[2]*se2h[3]+cse13rd)+(1-prev[12])*((1-sp[1])*(1-sp[2])*(1-sp[3])+csp13rd)
prob12[2,2,1] <- prev[12]*(se2h[1]*se2h[2]*(1-se2h[3])-cse13rd)+(1-prev[12])*((1-sp[1])*(1-sp[2])*sp[3]-csp13rd)
prob12[2,1,2] <- prev[12]*(se2h[1]*(1-se2h[2])*se2h[3]+cse13rd)+(1-prev[12])*((1-sp[1])*sp[2]*(1-sp[3])+csp13rd)
prob12[2,1,1] <- prev[12]*(se2h[1]*(1-se2h[2])*(1-se2h[3])-cse13rd)+(1-prev[12])*((1-sp[1])*sp[2]*sp[3]-csp13rd)
prob12[1,2,2] <- prev[12]*((1-se2h[1])*se2h[2]*se2h[3]-cse13rd)+(1-prev[12])*(sp[1]*(1-sp[2])*(1-sp[3])-csp13rd)
prob12[1,2,1] <- prev[12]*((1-se2h[1])*se2h[2]*(1-se2h[3])+cse13rd)+(1-prev[12])*(sp[1]*(1-sp[2])*sp[3]+csp13rd)
prob12[1,1,2] <- prev[12]*((1-se2h[1])*(1-se2h[2])*se2h[3]-cse13rd)+(1-prev[12])*(sp[1]*sp[2]*(1-sp[3])-csp13rd)
prob12[1,1,1] <- prev[12]*((1-se2h[1])*(1-se2h[2])*(1-se2h[3])+cse13rd)+(1-prev[12])*(sp[1]*sp[2]*sp[3]+csp13rd)

# pop13 – site D
prob13[2,2,2] <- prev[13]*(se2h[1]*se2h[2]*se2h[3]+cse13rd)+(1-prev[13])*((1-sp[1])*(1-sp[2])*(1-sp[3])+csp13rd)
prob13[2,2,1] <- prev[13]*(se2h[1]*se2h[2]*(1-se2h[3])-cse13rd)+(1-prev[13])*((1-sp[1])*(1-sp[2])*sp[3]-csp13rd)
prob13[2,1,2] <- prev[13]*(se2h[1]*(1-se2h[2])*se2h[3]+cse13rd)+(1-prev[13])*((1-sp[1])*sp[2]*(1-sp[3])+csp13rd)
prob13[2,1,1] <- prev[13]*(se2h[1]*(1-se2h[2])*(1-se2h[3])-cse13rd)+(1-prev[13])*((1-sp[1])*sp[2]*sp[3]-csp13rd)
prob13[1,2,2] <- prev[13]*((1-se2h[1])*se2h[2]*se2h[3]-cse13rd)+(1-prev[13])*(sp[1]*(1-sp[2])*(1-sp[3])-csp13rd)
prob13[1,2,1] <- prev[13]*((1-se2h[1])*se2h[2]*(1-se2h[3])+cse13rd)+(1-prev[13])*(sp[1]*(1-sp[2])*sp[3]+csp13rd)
prob13[1,1,2] <- prev[13]*((1-se2h[1])*(1-se2h[2])*se2h[3]-cse13rd)+(1-prev[13])*(sp[1]*sp[2]*(1-sp[3])-csp13rd)
prob13[1,1,1] <- prev[13]*((1-se2h[1])*(1-se2h[2])*(1-se2h[3])+cse13rd)+(1-prev[13])*(sp[1]*sp[2]*sp[3]+csp13rd)

## priors
# Prevalences
prev[1] ~ dbeta(0.5, 0.5)
prev[2] ~ dbeta(0.5, 0.5)
prev[3] ~ dbeta(0.5, 0.5)
prev[4] ~ dbeta(0.5, 0.5)
prev[5] ~ dbeta(0.5, 0.5)
prev[6] ~ dbeta(0.5, 0.5)
prev[7] ~ dbeta(0.5, 0.5)
prev[8] ~ dbeta(0.5, 0.5)
prev[9] ~ dbeta(0.5, 0.5)
prev[10] ~ dbeta(0.5, 0.5)
prev[11] ~ dbeta(0.5, 0.5)
prev[12] ~ dbeta(0.5, 0.5)
prev[13] ~ dbeta(0.5, 0.5)

# Se & Sp
# Low prevalence populations
se[1] ~ dbeta(4.071056, 1.856126)T(1-sp[1], )        # Se gross pathology WB LOW
sp[1] ~ dbeta(9.527001, 1.477224)                    # Sp gross pathology WB LOW
se[2] ~ dbeta(4.981778, 1.507261)T(1-sp[2], )        # Se PCR IS6110 WB LOW
sp[2] ~ dbeta(77.47122, 6.141608)                    # Sp PCR IS6110 Wb & cervids LOW & HIGH
```

```
  se[3] ~ dbeta(3.101771, 2.315743)T(1-sp[3], )          # Se bacteriological culture WB LOW
  sp[3] ~ dbeta(34.45668, 2.394028)                      # Sp bacteriological culture WB & cervids LOW & HIGH
  se2[1] ~ dbeta(7.81764, 2.758008)T(1-sp[1], )          # Se gross pathology cervids LOW
  se2[2] ~ dbeta(4.981778, 1.507261)T(1-sp[2], )         # Se PCR IS6110 cervids LOW
  se2[3] ~ dbeta(2.506658, 2.073236)T(1-sp2[1], )        # Se bacteriological culture cervids LOW
  sp2[1] ~ dbeta(34.45668, 2.394028)                     # Sp bacteriological culture cervids LOW & HIGH

# High prevalence populations
  seh[1] ~ dbeta(4.071056, 1.856126)T(1-sp[1], )         # Se gross pathology WB HIGH
  seh[2] ~ dbeta(4.981778, 1.507261)T(1-sp[2], )         # Se PCR IS6110 WB HIGH
  seh[3] ~ dbeta(3.101771, 2.315743)T(1-sp[3], )         # Se bacteriological culture WB HIGH
  se2h[1] ~ dbeta(7.81764, 2.758008)T(1-sp[1], )         # Se gross pathology cervids HIGH
  se2h[2] ~ dbeta(4.981778, 1.507261)T(1-sp[2], )        # Se PCR IS6110 cervids HIGH
  se2h[3] ~ dbeta(2.506658, 2.073236)T(1-sp2[1], )       # Se bacteriological culture cervids HIGH

  ## Covariances
  cse13wb ~ dunif((se[1]-1)*(1-se[3]), min(se[1], se[3]) - se[1]*se[3])
  csp13wb ~ dunif((sp[1]-1)*(1-sp[3]), min(sp[1], sp[3]) - sp[1]*sp[3])
  cse13rd ~ dunif((se2[1]-1)*(1-se2[3]), min(se2[1], se2[3]) - se2[1]*se2[3])
  csp13rd ~ dunif((sp[1]-1)*(1-sp[3]), min(sp[1], sp[3]) - sp[1]*sp[3])

  #data# pop_1, pop_2, pop_3, pop_4, pop_5, pop_6, pop_7, pop_8, pop_9, pop_10, pop_11, pop_12, pop_13, N_1, N_2, N_3, N_4, N_5, N_6,
N_7, N_8, N_9, N_10, N_11, N_12, N_13

  #monitor# prev, se, sp, seh, se2, sp2, se2h, cse13wb, csp13wb, cse23wb, csp23wb, cse13rd, csp13rd, cse23rd, csp23rd

#inits# prev, se, sp, seh, se2, sp2, se2h, cse13wb, csp13wb, cse23wb, csp23wb, cse13rd, csp13rd, cse23rd, csp23rd
}
")
```

Code S3. Code for the selected Hui-Walter model including the covariance between Test 2-Test 3. Code for Model 3.

```
model3 <- c("model{
 pop_1 ~ dmulti(prob1, N_1)
 pop_2 ~ dmulti(prob2, N_2)
 pop_3 ~ dmulti(prob3, N_3)
 pop_4 ~ dmulti(prob4, N_4)
 pop_5 ~ dmulti(prob5, N_5)
 pop_6 ~ dmulti(prob6, N_6)
 #pop_7 ~ dmulti(prob7, N_7)
 pop_8 ~ dmulti(prob8, N_8)
 pop_9 ~ dmulti(prob9, N_9)
 pop_10 ~ dmulti(prob10, N_10)
 pop_11 ~ dmulti(prob11, N_11)
 pop_12 ~ dmulti(prob12, N_12)
 pop_13 ~ dmulti(prob13, N_13)

 # wild boar
 # pop1 – site A
 prob1[2,2,2] <- prev[1]*(se[1]*se[2]*se[3]+cse23wb)+(1-prev[1])*((1-sp[1])*(1-sp[2])*(1-sp[3])+csp23wb)
 prob1[2,2,1] <- prev[1]*(se[1]*se[2]*(1-se[3])-cse23wb)+(1-prev[1])*((1-sp[1])*(1-sp[2])*sp[3]-csp23wb)
 prob1[2,1,2] <- prev[1]*(se[1]*(1-se[2])*se[3]-cse23wb)+(1-prev[1])*((1-sp[1])*sp[2]*(1-sp[3])-csp23wb)
 prob1[2,1,1] <- prev[1]*(se[1]*(1-se[2])*(1-se[3])+cse23wb)+(1-prev[1])*((1-sp[1])*sp[2]*sp[3]+csp23wb)
 prob1[1,2,2] <- prev[1]*((1-se[1])*se[2]*se[3]+cse23wb)+(1-prev[1])*(sp[1]*(1-sp[2])*(1-sp[3])+csp23wb)
 prob1[1,2,1] <- prev[1]*((1-se[1])*se[2]*(1-se[3])-cse23wb)+(1-prev[1])*(sp[1]*(1-sp[2])*sp[3]-csp23wb)
 prob1[1,1,2] <- prev[1]*((1-se[1])*(1-se[2])*se[3]-cse23wb)+(1-prev[1])*(sp[1]*sp[2]*(1-sp[3])-csp23wb)
 prob1[1,1,1] <- prev[1]*((1-se[1])*(1-se[2])*(1-se[3])+cse23wb)+(1-prev[1])*(sp[1]*sp[2]*sp[3]+csp23wb)

 # pop2 – site B
 prob2[2,2,2] <- prev[2]*(se[1]*se[2]*se[3]+cse23wb)+(1-prev[2])*((1-sp[1])*(1-sp[2])*(1-sp[3])+csp23wb)
 prob2[2,2,1] <- prev[2]*(se[1]*se[2]*(1-se[3])-cse23wb)+(1-prev[2])*((1-sp[1])*(1-sp[2])*sp[3]-csp23wb)
 prob2[2,1,2] <- prev[2]*(se[1]*(1-se[2])*se[3]-cse23wb)+(1-prev[2])*((1-sp[1])*sp[2]*(1-sp[3])-csp23wb)
 prob2[2,1,1] <- prev[2]*(se[1]*(1-se[2])*(1-se[3])+cse23wb)+(1-prev[2])*((1-sp[1])*sp[2]*sp[3]+csp23wb)
 prob2[1,2,2] <- prev[2]*((1-se[1])*se[2]*se[3]+cse23wb)+(1-prev[2])*(sp[1]*(1-sp[2])*(1-sp[3])+csp23wb)
 prob2[1,2,1] <- prev[2]*((1-se[1])*se[2]*(1-se[3])-cse23wb)+(1-prev[2])*(sp[1]*(1-sp[2])*sp[3]-csp23wb)
 prob2[1,1,2] <- prev[2]*((1-se[1])*(1-se[2])*se[3]-cse23wb)+(1-prev[2])*(sp[1]*sp[2]*(1-sp[3])-csp23wb)
 prob2[1,1,1] <- prev[2]*((1-se[1])*(1-se[2])*(1-se[3])+cse23wb)+(1-prev[2])*(sp[1]*sp[2]*sp[3]+csp23wb)

 # pop3 – site C
 prob3[2,2,2] <- prev[3]*(se[1]*se[2]*se[3]+cse23wb)+(1-prev[3])*((1-sp[1])*(1-sp[2])*(1-sp[3])+csp23wb)
 prob3[2,2,1] <- prev[3]*(se[1]*se[2]*(1-se[3])-cse23wb)+(1-prev[3])*((1-sp[1])*(1-sp[2])*sp[3]-csp23wb)
 prob3[2,1,2] <- prev[3]*(se[1]*(1-se[2])*se[3]-cse23wb)+(1-prev[3])*((1-sp[1])*sp[2]*(1-sp[3])-csp23wb)
 prob3[2,1,1] <- prev[3]*(se[1]*(1-se[2])*(1-se[3])+cse23wb)+(1-prev[3])*((1-sp[1])*sp[2]*sp[3]+csp23wb)
 prob3[1,2,2] <- prev[3]*((1-se[1])*se[2]*se[3]+cse23wb)+(1-prev[3])*(sp[1]*(1-sp[2])*(1-sp[3])+csp23wb)
 prob3[1,2,1] <- prev[3]*((1-se[1])*se[2]*(1-se[3])-cse23wb)+(1-prev[3])*(sp[1]*(1-sp[2])*sp[3]-csp23wb)
 prob3[1,1,2] <- prev[3]*((1-se[1])*(1-se[2])*se[3]-cse23wb)+(1-prev[3])*(sp[1]*sp[2]*(1-sp[3])-csp23wb)
 prob3[1,1,1] <- prev[3]*((1-se[1])*(1-se[2])*(1-se[3])+cse23wb)+(1-prev[3])*(sp[1]*sp[2]*sp[3]+csp23wb)

 # pop4 – site D
 prob4[2,2,2] <- prev[4]*(se[1]*se[2]*se[3]+cse23wb)+(1-prev[4])*((1-sp[1])*(1-sp[2])*(1-sp[3])+csp23wb)
 prob4[2,2,1] <- prev[4]*(se[1]*se[2]*(1-se[3])-cse23wb)+(1-prev[4])*((1-sp[1])*(1-sp[2])*sp[3]-csp23wb)
 prob4[2,1,2] <- prev[4]*(se[1]*(1-se[2])*se[3]-cse23wb)+(1-prev[4])*((1-sp[1])*sp[2]*(1-sp[3])-csp23wb)
 prob4[2,1,1] <- prev[4]*(se[1]*(1-se[2])*(1-se[3])+cse23wb)+(1-prev[4])*((1-sp[1])*sp[2]*sp[3]+csp23wb)
 prob4[1,2,2] <- prev[4]*((1-se[1])*se[2]*se[3]+cse23wb)+(1-prev[4])*(sp[1]*(1-sp[2])*(1-sp[3])+csp23wb)
 prob4[1,2,1] <- prev[4]*((1-se[1])*se[2]*(1-se[3])-cse23wb)+(1-prev[4])*(sp[1]*(1-sp[2])*sp[3]-csp23wb)
 prob4[1,1,2] <- prev[4]*((1-se[1])*(1-se[2])*se[3]-cse23wb)+(1-prev[4])*(sp[1]*sp[2]*(1-sp[3])-csp23wb)
```

```
prob4[1,1,1] <- prev[4]*((1-se[1])*(1-se[2])*(1-se[3])+cse23wb)+(1-prev[4])*(sp[1]*sp[2]*sp[3]+csp23wb)

# pop5 – site E
prob5[2,2,2] <- prev[5]*(se[1]*se[2]*se[3]+cse23wb)+(1-prev[5])*((1-sp[1])*(1-sp[2])*(1-sp[3])+csp23wb)
prob5[2,2,1] <- prev[5]*(se[1]*se[2]*(1-se[3])-cse23wb)+(1-prev[5])*((1-sp[1])*(1-sp[2])*sp[3]-csp23wb)
prob5[2,1,2] <- prev[5]*(se[1]*(1-se[2])*se[3]-cse23wb)+(1-prev[5])*((1-sp[1])*sp[2]*(1-sp[3])-csp23wb)
prob5[2,1,1] <- prev[5]*(se[1]*(1-se[2])*(1-se[3])+cse23wb)+(1-prev[5])*((1-sp[1])*sp[2]*sp[3]+csp23wb)
prob5[1,2,2] <- prev[5]*((1-se[1])*se[2]*se[3]+cse23wb)+(1-prev[5])*(sp[1]*(1-sp[2])*(1-sp[3])+csp23wb)
prob5[1,2,1] <- prev[5]*((1-se[1])*se[2]*(1-se[3])-cse23wb)+(1-prev[5])*(sp[1]*(1-sp[2])*sp[3]-csp23wb)
prob5[1,1,2] <- prev[5]*((1-se[1])*(1-se[2])*se[3]-cse23wb)+(1-prev[5])*(sp[1]*sp[2]*(1-sp[3])-csp23wb)
prob5[1,1,1] <- prev[5]*((1-se[1])*(1-se[2])*(1-se[3])+cse23wb)+(1-prev[5])*(sp[1]*sp[2]*sp[3]+csp23wb)

# pop6 – site F
prob6[2,2,2] <- prev[6]*(se[1]*se[2]*se[3]+cse23wb)+(1-prev[6])*((1-sp[1])*(1-sp[2])*(1-sp[3])+csp23wb)
prob6[2,2,1] <- prev[6]*(se[1]*se[2]*(1-se[3])-cse23wb)+(1-prev[6])*((1-sp[1])*(1-sp[2])*sp[3]-csp23wb)
prob6[2,1,2] <- prev[6]*(se[1]*(1-se[2])*se[3]-cse23wb)+(1-prev[6])*((1-sp[1])*sp[2]*(1-sp[3])-csp23wb)
prob6[2,1,1] <- prev[6]*(se[1]*(1-se[2])*(1-se[3])+cse23wb)+(1-prev[6])*((1-sp[1])*sp[2]*sp[3]+csp23wb)
prob6[1,2,2] <- prev[6]*((1-se[1])*se[2]*se[3]+cse23wb)+(1-prev[6])*(sp[1]*(1-sp[2])*(1-sp[3])+csp23wb)
prob6[1,2,1] <- prev[6]*((1-se[1])*se[2]*(1-se[3])-cse23wb)+(1-prev[6])*(sp[1]*(1-sp[2])*sp[3]-csp23wb)
prob6[1,1,2] <- prev[6]*((1-se[1])*(1-se[2])*se[3]-cse23wb)+(1-prev[6])*(sp[1]*sp[2]*(1-sp[3])-csp23wb)
prob6[1,1,1] <- prev[6]*((1-se[1])*(1-se[2])*(1-se[3])+cse23wb)+(1-prev[6])*(sp[1]*sp[2]*sp[3]+csp23wb)

# red deer
# pop7 – site A
#prob7[2,2,2] <- prev[7]*(se2[1]*se2[2]*se2[3]+cse23rd)+(1-prev[7])*((1-sp2[1])*(1-sp2[2])*(1-sp2[3])+csp23rd)
#prob7[2,2,1] <- prev[7]*(se2[1]*se2[2]*(1-se2[3])-cse23rd)+(1-prev[7])*((1-sp2[1])*(1-sp2[2])*sp2[3]-csp23rd)
#prob7[2,1,2] <- prev[7]*(se2[1]*(1-se2[2])*se2[3]-cse23rd)+(1-prev[7])*((1-sp2[1])*sp2[2]*(1-sp2[3])-csp23rd)
#prob7[2,1,1] <- prev[7]*(se2[1]*(1-se2[2])*(1-se2[3])+cse23rd)+(1-prev[7])*((1-sp2[1])*sp2[2]*sp2[3]+csp23rd)
#prob7[1,2,2] <- prev[7]*((1-se2[1])*se2[2]*se2[3]+cse23rd)+(1-prev[7])*(sp2[1]*(1-sp2[2])*(1-sp2[3])+csp23rd)
#prob7[1,2,1] <- prev[7]*((1-se2[1])*se2[2]*(1-se2[3])-cse23rd)+(1-prev[7])*(sp2[1]*(1-sp2[2])*sp2[3]-csp23rd)
#prob7[1,1,2] <- prev[7]*((1-se2[1])*(1-se2[2])*se2[3]-cse23rd)+(1-prev[7])*(sp2[1]*sp2[2]*(1-sp2[3])-csp23rd)
#prob7[1,1,1] <- prev[7]*((1-se2[1])*(1-se2[2])*(1-se2[3])+cse23rd)+(1-prev[7])*(sp2[1]*sp2[2]*sp2[3]+csp23rd)

# pop8 – site C
prob8[2,2,2] <- prev[8]*(se2[1]*se2[2]*se2[3]+cse23rd)+(1-prev[8])*((1-sp2[1])*(1-sp2[2])*(1-sp2[3])+csp23rd)
prob8[2,2,1] <- prev[8]*(se2[1]*se2[2]*(1-se2[3])-cse23rd)+(1-prev[8])*((1-sp2[1])*(1-sp2[2])*sp2[3]-csp23rd)
prob8[2,1,2] <- prev[8]*(se2[1]*(1-se2[2])*se2[3]-cse23rd)+(1-prev[8])*((1-sp2[1])*sp2[2]*(1-sp2[3])-csp23rd)
prob8[2,1,1] <- prev[8]*(se2[1]*(1-se2[2])*(1-se2[3])+cse23rd)+(1-prev[8])*((1-sp2[1])*sp2[2]*sp2[3]+csp23rd)
prob8[1,2,2] <- prev[8]*((1-se2[1])*se2[2]*se2[3]+cse23rd)+(1-prev[8])*(sp2[1]*(1-sp2[2])*(1-sp2[3])+csp23rd)
prob8[1,2,1] <- prev[8]*((1-se2[1])*se2[2]*(1-se2[3])-cse23rd)+(1-prev[8])*(sp2[1]*(1-sp2[2])*sp2[3]-csp23rd)
prob8[1,1,2] <- prev[8]*((1-se2[1])*(1-se2[2])*se2[3]-cse23rd)+(1-prev[8])*(sp2[1]*sp2[2]*(1-sp2[3])-csp23rd)
prob8[1,1,1] <- prev[8]*((1-se2[1])*(1-se2[2])*(1-se2[3])+cse23rd)+(1-prev[8])*(sp2[1]*sp2[2]*sp2[3]+csp23rd)

# pop9 – site D
prob9[2,2,2] <- prev[9]*(se2[1]*se2[2]*se2[3]+cse23rd)+(1-prev[9])*((1-sp2[1])*(1-sp2[2])*(1-sp2[3])+csp23rd)
prob9[2,2,1] <- prev[9]*(se2[1]*se2[2]*(1-se2[3])-cse23rd)+(1-prev[9])*((1-sp2[1])*(1-sp2[2])*sp2[3]-csp23rd)
prob9[2,1,2] <- prev[9]*(se2[1]*(1-se2[2])*se2[3]-cse23rd)+(1-prev[9])*((1-sp2[1])*sp2[2]*(1-sp2[3])-csp23rd)
prob9[2,1,1] <- prev[9]*(se2[1]*(1-se2[2])*(1-se2[3])+cse23rd)+(1-prev[9])*((1-sp2[1])*sp2[2]*sp2[3]+csp23rd)
prob9[1,2,2] <- prev[9]*((1-se2[1])*se2[2]*se2[3]+cse23rd)+(1-prev[9])*(sp2[1]*(1-sp2[2])*(1-sp2[3])+csp23rd)
prob9[1,2,1] <- prev[9]*((1-se2[1])*se2[2]*(1-se2[3])-cse23rd)+(1-prev[9])*(sp2[1]*(1-sp2[2])*sp2[3]-csp23rd)
prob9[1,1,2] <- prev[9]*((1-se2[1])*(1-se2[2])*se2[3]-cse23rd)+(1-prev[9])*(sp2[1]*sp2[2]*(1-sp2[3])-csp23rd)
prob9[1,1,1] <- prev[9]*((1-se2[1])*(1-se2[2])*(1-se2[3])+cse23rd)+(1-prev[9])*(sp2[1]*sp2[2]*sp2[3]+csp23rd)

# pop10 – site E
prob10[2,2,2] <- prev[10]*(se2[1]*se2[2]*se2[3]+cse23rd)+(1-prev[10])*((1-sp2[1])*(1-sp2[2])*(1-sp2[3])+csp23rd)
prob10[2,2,1] <- prev[10]*(se2[1]*se2[2]*(1-se2[3])-cse23rd)+(1-prev[10])*((1-sp2[1])*(1-sp2[2])*sp2[3]-csp23rd)
prob10[2,1,2] <- prev[10]*(se2[1]*(1-se2[2])*se2[3]-cse23rd)+(1-prev[10])*((1-sp2[1])*sp2[2]*(1-sp2[3])-csp23rd)
prob10[2,1,1] <- prev[10]*(se2[1]*(1-se2[2])*(1-se2[3])+cse23rd)+(1-prev[10])*((1-sp2[1])*sp2[2]*sp2[3]+csp23rd)
```

```
prob10[1,2,2] <- prev[10]*((1-se2[1])*se2[2]*se2[3]+cse23rd)+(1-prev[10])*(sp2[1]*(1-sp2[2])*(1-sp2[3])+csp23rd)
prob10[1,2,1] <- prev[10]*((1-se2[1])*se2[2]*(1-se2[3])-cse23rd)+(1-prev[10])*(sp2[1]*(1-sp2[2])*sp2[3]-csp23rd)
prob10[1,1,2] <- prev[10]*((1-se2[1])*(1-se2[2])*se2[3]-cse23rd)+(1-prev[10])*(sp2[1]*sp2[2]*(1-sp2[3])-csp23rd)
prob10[1,1,1] <- prev[10]*((1-se2[1])*(1-se2[2])*(1-se2[3])+cse23rd)+(1-prev[10])*(sp2[1]*sp2[2]*sp2[3]+csp23rd)

# pop11 – site F
prob11[2,2,2] <- prev[11]*(se2[1]*se2[2]*se2[3]+cse23rd)+(1-prev[11])*((1-sp2[1])*(1-sp2[2])*(1-sp2[3])+csp23rd)
prob11[2,2,1] <- prev[11]*(se2[1]*se2[2]*(1-se2[3])-cse23rd)+(1-prev[11])*((1-sp2[1])*(1-sp2[2])*sp2[3]-csp23rd)
prob11[2,1,2] <- prev[11]*(se2[1]*(1-se2[2])*se2[3]-cse23rd)+(1-prev[11])*((1-sp2[1])*sp2[2]*(1-sp2[3])-csp23rd)
prob11[2,1,1] <- prev[11]*(se2[1]*(1-se2[2])*(1-se2[3])+cse23rd)+(1-prev[11])*((1-sp2[1])*sp2[2]*sp2[3]+csp23rd)
prob11[1,2,2] <- prev[11]*((1-se2[1])*se2[2]*se2[3]+cse23rd)+(1-prev[11])*(sp2[1]*(1-sp2[2])*(1-sp2[3])+csp23rd)
prob11[1,2,1] <- prev[11]*((1-se2[1])*se2[2]*(1-se2[3])-cse23rd)+(1-prev[11])*(sp2[1]*(1-sp2[2])*sp2[3]-csp23rd)
prob11[1,1,2] <- prev[11]*((1-se2[1])*(1-se2[2])*se2[3]-cse23rd)+(1-prev[11])*(sp2[1]*sp2[2]*(1-sp2[3])-csp23rd)
prob11[1,1,1] <- prev[11]*((1-se2[1])*(1-se2[2])*(1-se2[3])+cse23rd)+(1-prev[11])*(sp2[1]*sp2[2]*sp2[3]+csp23rd)

# fallow deer
# pop12 – site C
prob12[2,2,2] <- prev[12]*(se2[1]*se2[2]*se2[3]+cse23rd)+(1-prev[12])*((1-sp2[1])*(1-sp2[2])*(1-sp2[3])+csp23rd)
prob12[2,2,1] <- prev[12]*(se2[1]*se2[2]*(1-se2[3])-cse23rd)+(1-prev[12])*((1-sp2[1])*(1-sp2[2])*sp2[3]-csp23rd)
prob12[2,1,2] <- prev[12]*(se2[1]*(1-se2[2])*se2[3]-cse23rd)+(1-prev[12])*((1-sp2[1])*sp2[2]*(1-sp2[3])-csp23rd)
prob12[2,1,1] <- prev[12]*(se2[1]*(1-se2[2])*(1-se2[3])+cse23rd)+(1-prev[12])*((1-sp2[1])*sp2[2]*sp2[3]+csp23rd)
prob12[1,2,2] <- prev[12]*((1-se2[1])*se2[2]*se2[3]+cse23rd)+(1-prev[12])*(sp2[1]*(1-sp2[2])*(1-sp2[3])+csp23rd)
prob12[1,2,1] <- prev[12]*((1-se2[1])*se2[2]*(1-se2[3])-cse23rd)+(1-prev[12])*(sp2[1]*(1-sp2[2])*sp2[3]-csp23rd)
prob12[1,1,2] <- prev[12]*((1-se2[1])*(1-se2[2])*se2[3]-cse23rd)+(1-prev[12])*(sp2[1]*sp2[2]*(1-sp2[3])-csp23rd)
prob12[1,1,1] <- prev[12]*((1-se2[1])*(1-se2[2])*(1-se2[3])+cse23rd)+(1-prev[12])*(sp2[1]*sp2[2]*sp2[3]+csp23rd)

# pop13 – site D
prob13[2,2,2] <- prev[13]*(se2[1]*se2[2]*se2[3]+cse23rd)+(1-prev[13])*((1-sp2[1])*(1-sp2[2])*(1-sp2[3])+csp23rd)
prob13[2,2,1] <- prev[13]*(se2[1]*se2[2]*(1-se2[3])-cse23rd)+(1-prev[13])*((1-sp2[1])*(1-sp2[2])*sp2[3]-csp23rd)
prob13[2,1,2] <- prev[13]*(se2[1]*(1-se2[2])*se2[3]-cse23rd)+(1-prev[13])*((1-sp2[1])*sp2[2]*(1-sp2[3])-csp23rd)
prob13[2,1,1] <- prev[13]*(se2[1]*(1-se2[2])*(1-se2[3])+cse23rd)+(1-prev[13])*((1-sp2[1])*sp2[2]*sp2[3]+csp23rd)
prob13[1,2,2] <- prev[13]*((1-se2[1])*se2[2]*se2[3]+cse23rd)+(1-prev[13])*(sp2[1]*(1-sp2[2])*(1-sp2[3])+csp23rd)
prob13[1,2,1] <- prev[13]*((1-se2[1])*se2[2]*(1-se2[3])-cse23rd)+(1-prev[13])*(sp2[1]*(1-sp2[2])*sp2[3]-csp23rd)
prob13[1,1,2] <- prev[13]*((1-se2[1])*(1-se2[2])*se2[3]-cse23rd)+(1-prev[13])*(sp2[1]*sp2[2]*(1-sp2[3])-csp23rd)
prob13[1,1,1] <- prev[13]*((1-se2[1])*(1-se2[2])*(1-se2[3])+cse23rd)+(1-prev[13])*(sp2[1]*sp2[2]*sp2[3]+csp23rd)

## Priors
# Prevalences
prev[1] ~ dbeta(0.5, 0.5)
prev[2] ~ dbeta(0.5, 0.5)
prev[3] ~ dbeta(0.5, 0.5)
prev[4] ~ dbeta(0.5, 0.5)
prev[5] ~ dbeta(0.5, 0.5)
prev[6] ~ dbeta(0.5, 0.5)
prev[7] ~ dbeta(0.5, 0.5)
prev[8] ~ dbeta(0.5, 0.5)
prev[9] ~ dbeta(0.5, 0.5)
prev[10] ~ dbeta(0.5, 0.5)
prev[11] ~ dbeta(0.5, 0.5)
prev[12] ~ dbeta(0.5, 0.5)
prev[13] ~ dbeta(0.5, 0.5)

# Se & Sp
se[1] ~ dbeta(18.67196, 3.758051)T(1-sp[1], )          # Se gross pathology Wild boar
sp[1] ~ dbeta(237.1672, 5.084275)                      # Sp gross pathology Wild boar
se[2] ~ dbeta(26.66585, 5.852403)T(1-sp[2], )          # Se P22 ELISA Wild boar
sp[2] ~ dbeta(96.33597, 2.550178)                      # Sp P22 ELISA Wild boar
se[3] ~ dbeta(16.53371, 2.314131)T(1-sp[3], )          # Se bacteriology Wild boar
```

```
  sp[3] ~ dbeta(312.5549, 3.51254)                        # Sp bacteriology Wild boar
  se2[1] ~ dbeta(27.51271, 7.92072)T(1-sp2[1], )          # Se gross pathology Cervids
  sp2[1] ~ dbeta(75.38667, 3.538532)                      # Sp gross pathology Cervids
  se2[2] ~ dbeta(9.22462, 4.508076)T(1-sp2[2], )          # Se P22 ELISA Cervids
  sp2[2] ~ dbeta(62.13694, 1.617545)                      # Sp P22 ELISA Cervids
  se2[3] ~ dbeta(32.28187, 14.15261)T(1-sp2[3], )         # Se bacteriology Cervids
  sp2[3] ~ dbeta(312.5549, 3.51254)                       # Sp bacteriology Cervids

  # Covariances
  cse23wb ~ dunif((se[2]-1)*(1-se[3]), min(se[2], se[3]) - se[2]*se[3])
  csp23wb ~ dunif((sp[2]-1)*(1-sp[3]), min(sp[2], sp[3]) - sp[2]*sp[3])
  cse23rd ~ dunif((se2[2]-1)*(1-se2[3]), min(se2[2], se2[3]) - se2[2]*se2[3])
  csp23rd ~ dunif((sp2[2]-1)*(1-sp2[3]), min(sp2[2], sp2[3]) - sp2[2]*sp2[3])

  # Correlations
  cor23pwb <- cse23wb / sqrt(se[2]*(1-se[2])*se[3]*(1-se[3]))
  cor23nwb <- csp23wb / sqrt(sp[2]*(1-sp[2])*sp[3]*(1-sp[3]))
  cor23prd <- cse23rd / sqrt(se2[2]*(1-se2[2])*se2[3]*(1-se2[3]))
  cor23nrd <- csp23rd / sqrt(sp2[2]*(1-sp2[2])*sp2[3]*(1-sp2[3]))

  # parallel pathology-ELISA cervids
  se_par12<-1-((1-se2[1])*(1-se2[2]))
  sp_par12<-sp2[1]*sp2[2]

  # parallel culture-ELISA cervids
  se_par23<-1-((1-se2[2])*(1-se2[3]))
  sp_par23<-sp2[2]*sp2[3]

   #data# pop_1, pop_2, pop_3, pop_4, pop_5, pop_6, pop_8, pop_9, pop_10, pop_11, pop_12, pop_13, N_1, N_2, N_3, N_4, N_5, N_6, N_8,
N_9, N_10, N_11, N_12, N_13

  #monitor# prev, se, sp, se2, sp2, cse23wb, csp23wb, cse23rd, csp23rd, cor23pwb, cor23nwb, cor23prd, cor23nrd, se_par12, sp_par12, se_par23,
sp_par23

  #inits# prev, se, sp, se2, sp2, cse23wb, csp23wb, cse23rd, csp23rd
}
")
```

Table S3. Cross-tabulated test results by species and population. Test 1: gross pathology; Test 2: real-time PCR; Test 3: bacteriological culture.

Site A<br>Wild boar	T1-		T1+	
	T2-	T2+	T2-	T2-
T3-	86	4	0	0
T3+	0	0	0	0

Site B<br>Wild boar	T1-		T1+	
	T2-	T2+	T2-	T2-
T3-	36	0	0	0
T3+	1	0	0	0

Site C<br>Wild boar	T1-		T1+	
	T2-	T2+	T2-	T2+
T3-	29	2	29	2
T3+	1	0	1	0

Site D<br>Wild boar	T1-		T1+	
	T2-	T2+	T2-	T2+
T3-	3	0	3	0
T3+	0	0	0	0

Site E<br>Wild boar	T1-		T1+	
	T2-	T2+	T2-	T2+
T3-	28	1	0	0
T3+	0	0	0	0

Site F<br>Wild boar	T1-		T1+	
	T2-	T2+	T2-	T2+
T3-	5	0	0	0
T3+	1	0	6	3

Site A<br>Red deer	T1-		T1+	
	T2-	T2+	T2-	T2+
T3-	14	0	0	0
T3+	0	0	0	0

Site C<br>Red deer	T1-		T1+	
	T2-	T2+	T2-	T2+
T3-	8	0	0	1
T3+	0	0	1	0

Site D<br>Red deer	T1-		T1+	
	T2-	T2+	T2-	T2+
T3-	15	0	2	0
T3+	0	0	1	1

Site E<br>Red deer	T1-		T1+	
	T2-	T2+	T2-	T2+
T3-	22	0	0	0
T3+	0	0	0	0

Site F<br>Red deer	T1-		T1+	
	T2-	T2+	T2-	T2+
T3-	13	0	2	0
T3+	5	2	1	4

Site C<br>Fallow deer	T1-		T1+	
	T2-	T2+	T2-	T2+
T3-	2	0	0	0
T3+	0	0	1	0

Site D<br>Fallow deer	T1-		T1+	
	T2-	T2+	T2-	T2+
T3-	1	0	0	0
T3+	1	0	0	0

Fig S2. Diagnostic plots for Model 1: performance of the tests. Model 1 with diffuse priors from the bibliography for the Sensitivity and Specificity of all tests in wild boar and cervids.

Wild boar

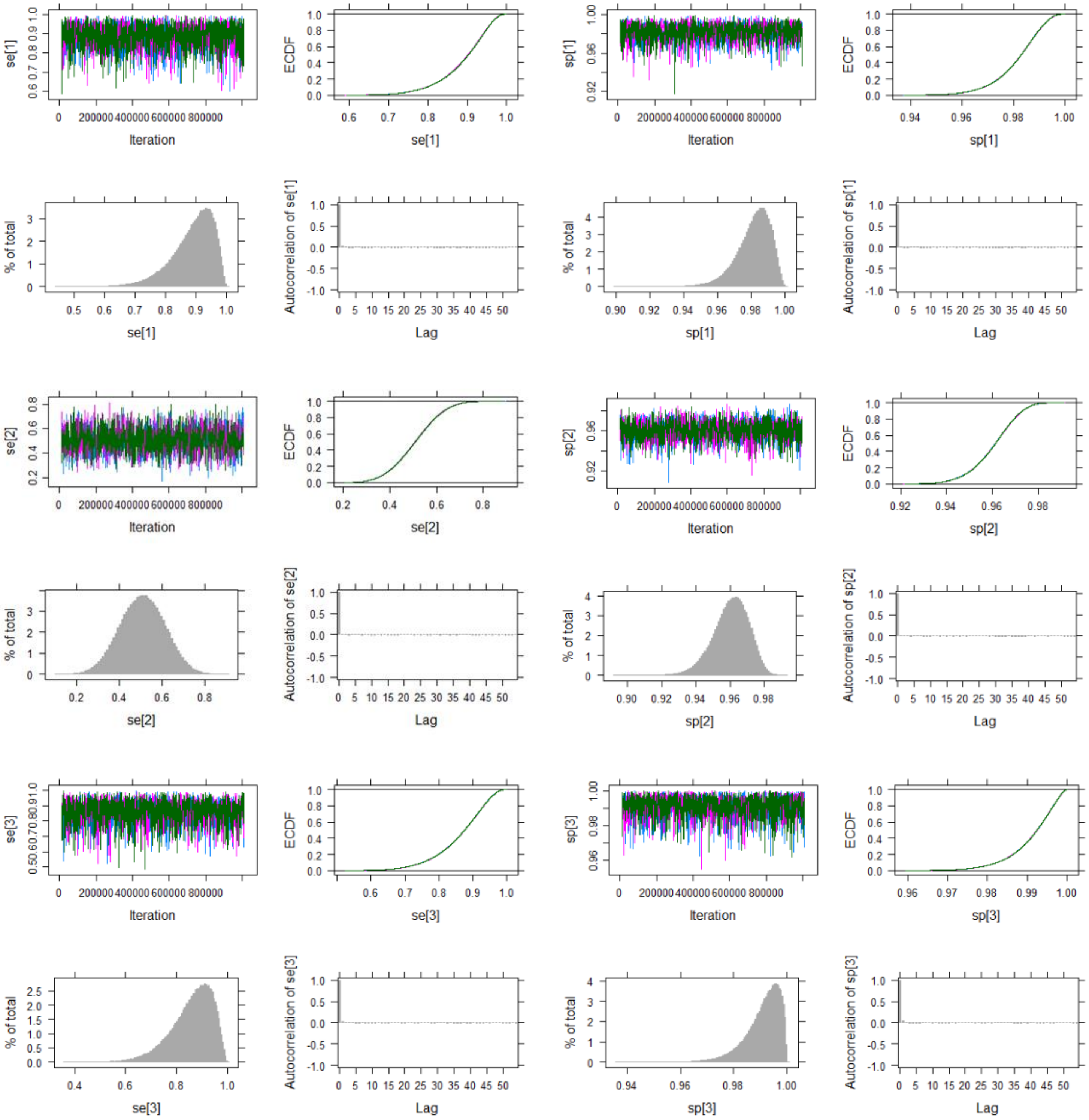

Cervids

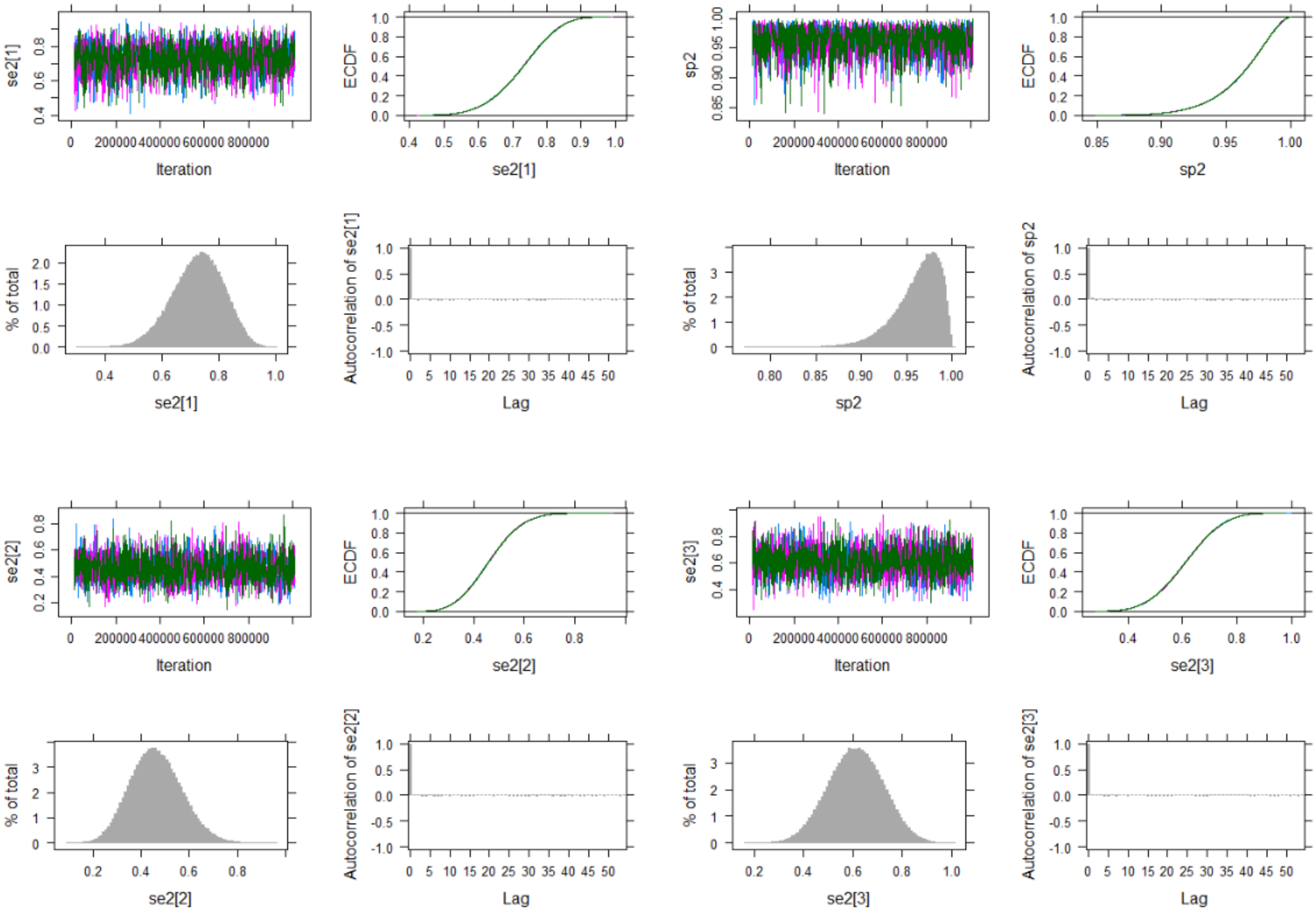

se2[1]
Iteration
ECDF
se2[1]
sp2
Iteration
ECDF
sp2
% of total
se2[1]
Autocorrelation of se2[1]
Lag
% of total
sp2
Autocorrelation of sp2
Lag
se2[2]
Iteration
ECDF
se2[2]
se2[3]
Iteration
ECDF
se2[3]
% of total
se2[2]
Autocorrelation of se2[2]
Lag
% of total
se2[3]
Autocorrelation of se2[3]
Lag

Fig S3. Diagnostic plots for Model 1: prevalence across populations. Prevalence across sites from Model 1 with diffuse priors from the bibliography for Se and Sp of all tests in wild boar and cervids.

Wild boar

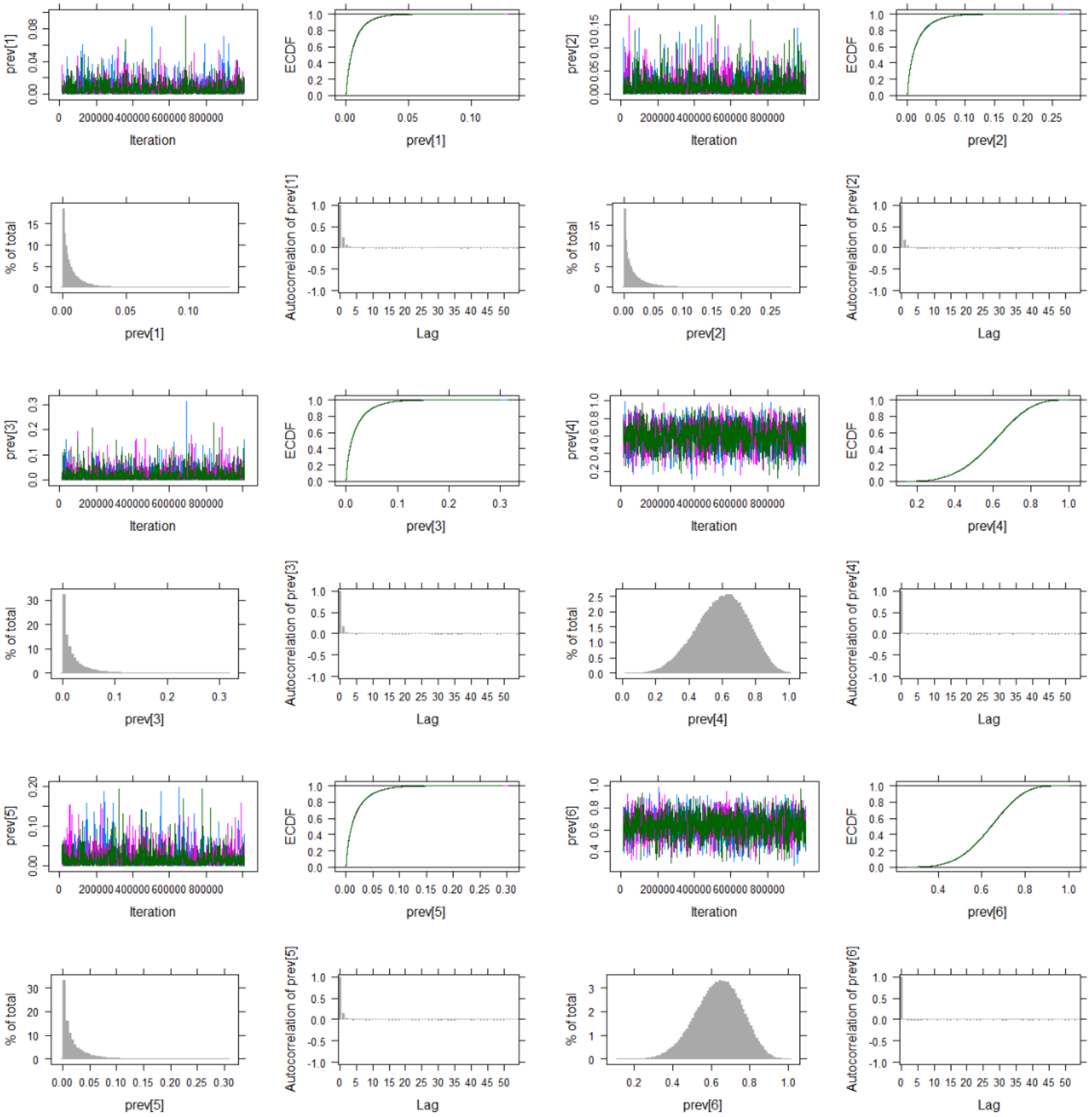

Red deer

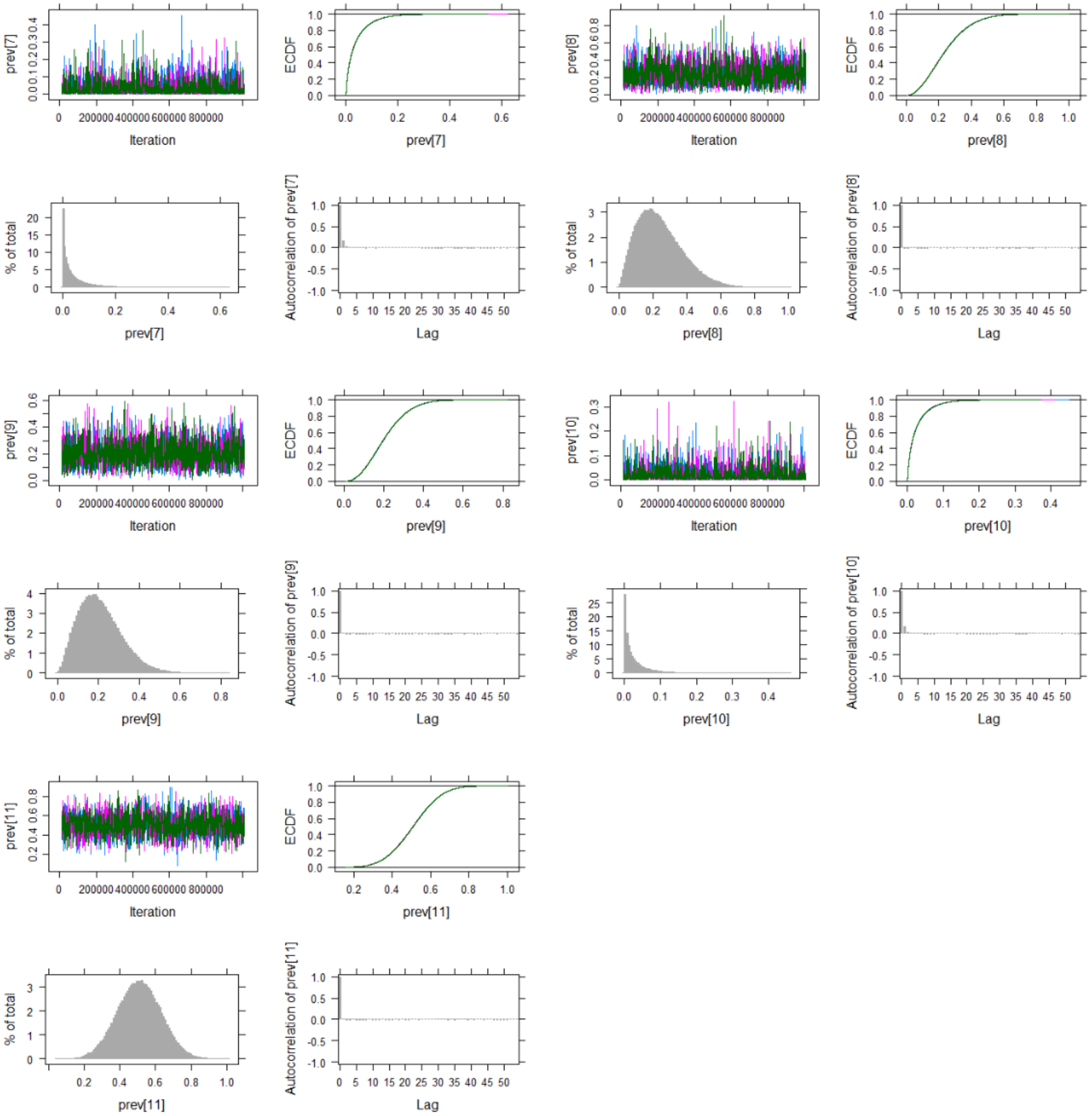
prev[7]
Iteration
ECDF
% of total
Autocorrelation of prev[7]
Lag
prev[8]
Autocorrelation of prev[8]
prev[9]
Autocorrelation of prev[9]
prev[10]
Autocorrelation of prev[10]
prev[11]
Autocorrelation of prev[11]

Fallow deer

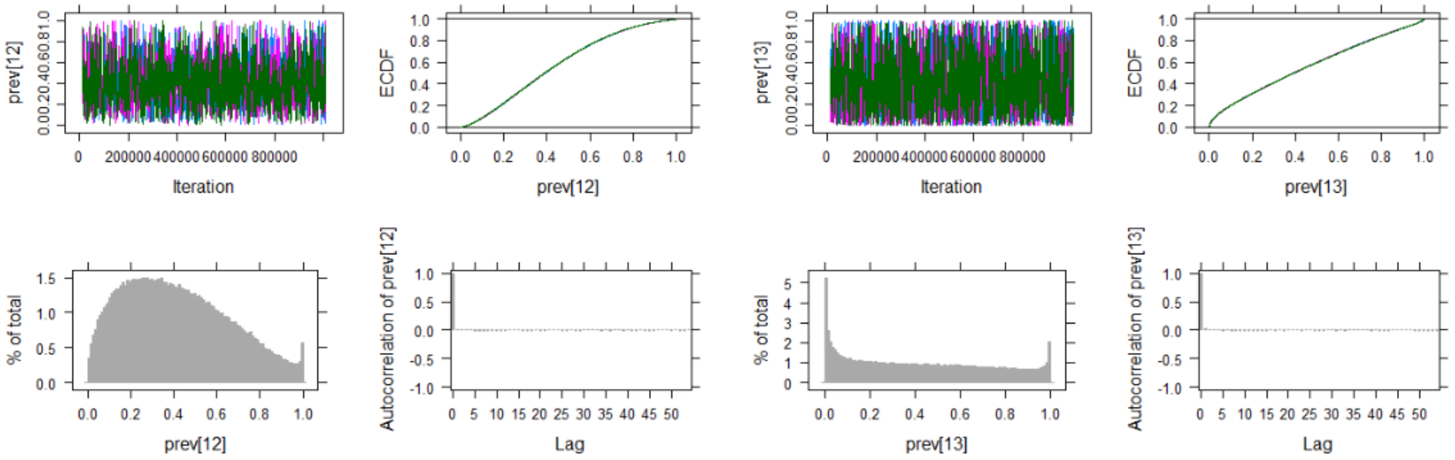
prev[12]
Iteration
ECDF
prev[12]
prev[13]
Iteration
ECDF
prev[13]
% of total
prev[12]
Autocorrelation of prev[12]
Lag
% of total
prev[13]
Autocorrelation of prev[13]
Lag

Fig S4. Plots of the sensitivity analysis for Models 1-3.

For every model, a leave-one-out sensitivity analysis was performed by running the models excluding one animal at a time from the dataset and comparing the posterior distribution of each parameter (in red, with mean and 95% confidence interval in black) with those obtained using the whole dataset (mean as dashed grey line). A sensitivity analysis of the priors was also performed for Model 1 by comparing estimates obtained using priors derived from the bibliography and minimally informative Jeffrey's priors.

Posterior estimates of the parameters (in red) when excluding one animal with the following combination of diagnostic test results: PPP – positive to all tests; PPN – positive to test 1 and test 2, negative to test 3; PNP – positive to test 1 and test3, negative to test 2; PNN – positive to test 1, negative to test 2 and test 3; NPP – negative to test 1, positive to test 2 and test 3; NPN – negative to test 1 and test 3, positive to test 2; NNP – negative to test 1 and test 2, positive to test 3; NNN – negative to all tests. Note that not all combinations of diagnostic test results are present in each dataset.

Model 1

Wild boar data removal – wild boar parameters

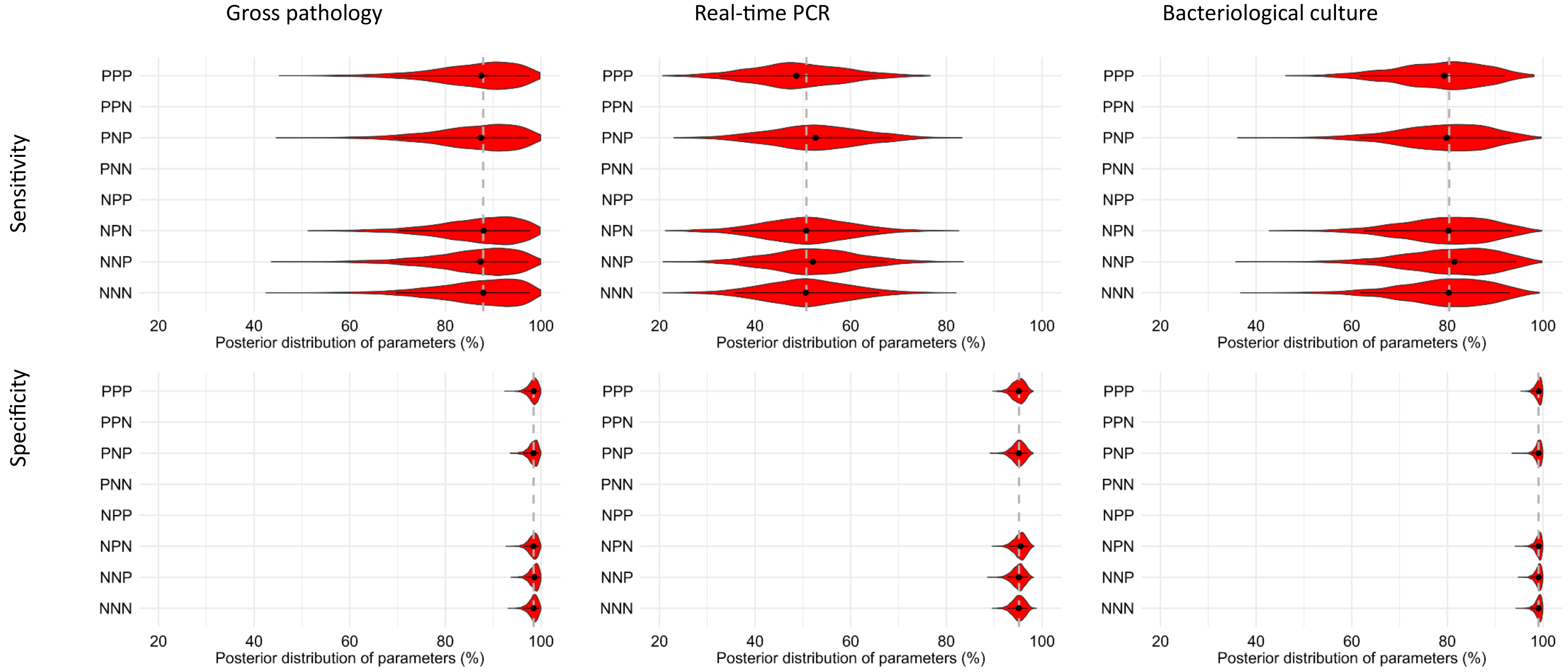

Model 1

Cervid data removal – cervid parameters

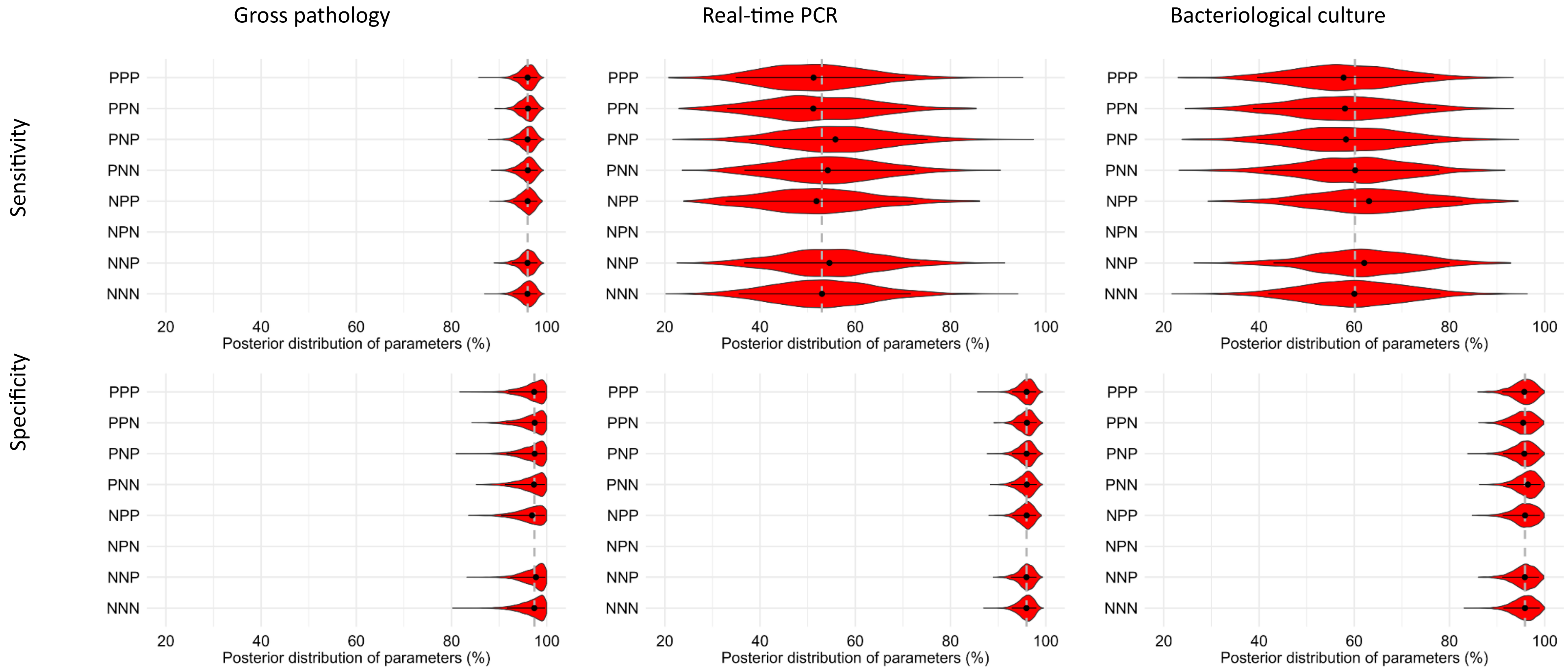

Model 1

Priors based on the bibliography vs Jeffrey's priors.

Wild boar parameters

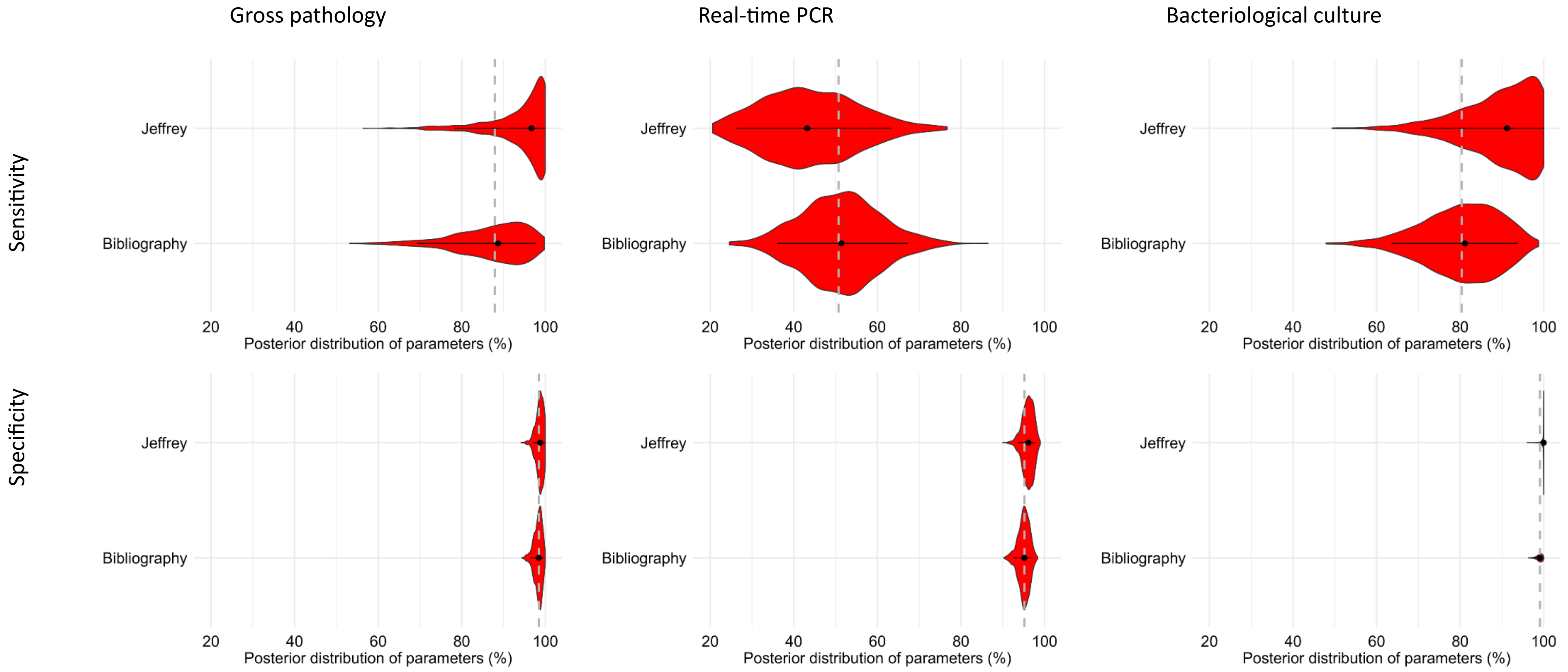

Model 1

Cervid parameters

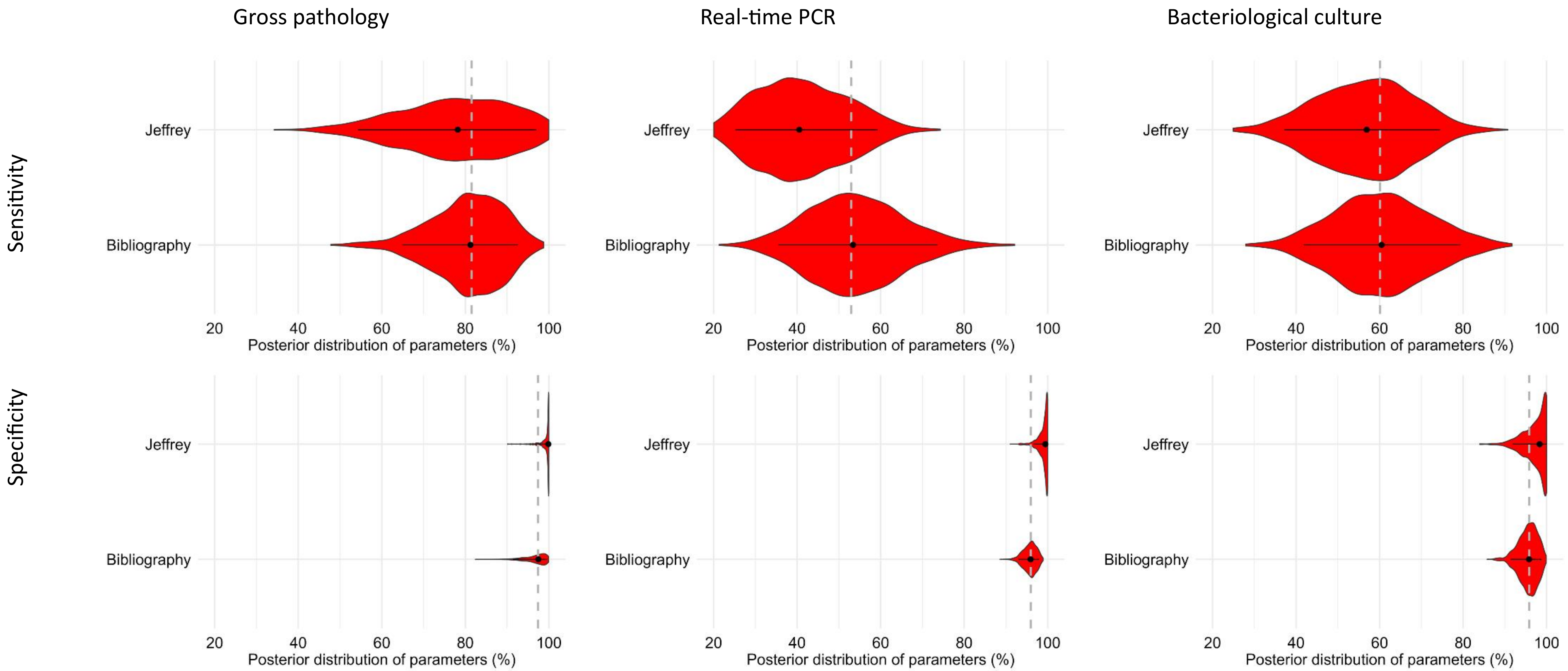

Model 2

Wild boar data removal from the pooled low prevalence group – wild boar Sensitivity parameters in the low prevalence group

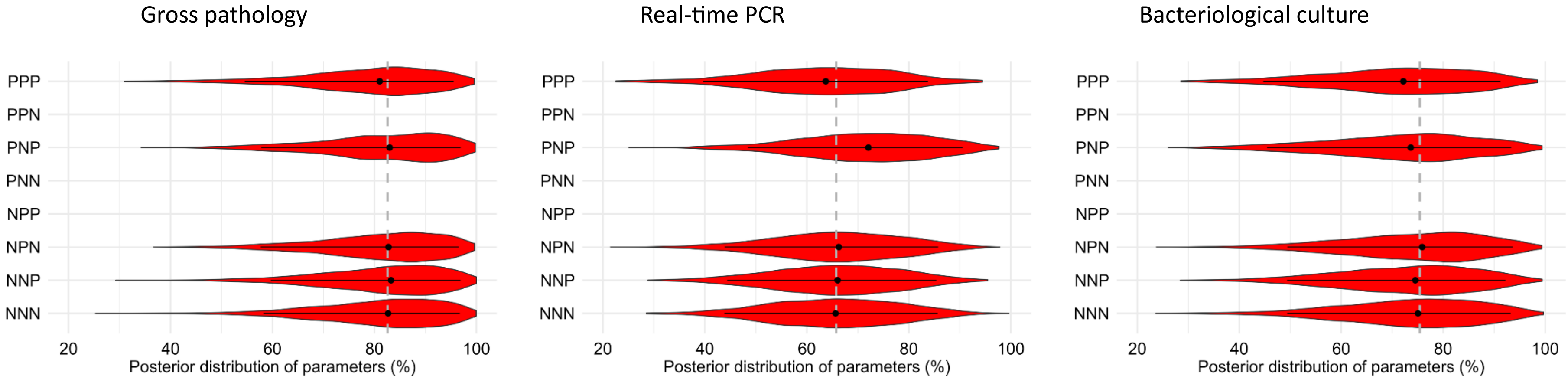

Cervid data removal from the pooled low prevalence group – cervid Sensitivity parameters in the low prevalence group

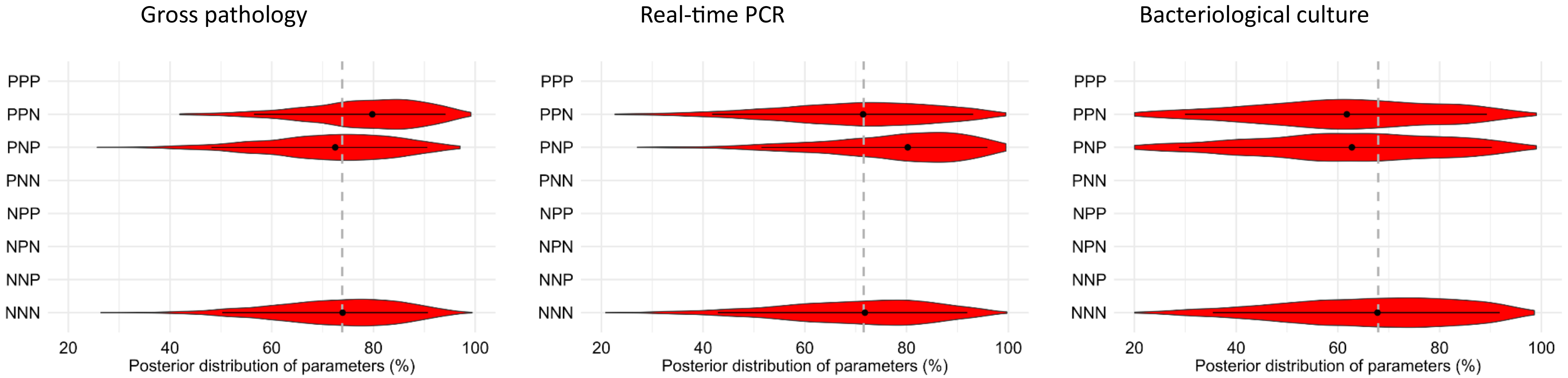

Model 2

Wild boar data removal from the pooled high prevalence group – wild boar Sensitivity parameters in the high prevalence group

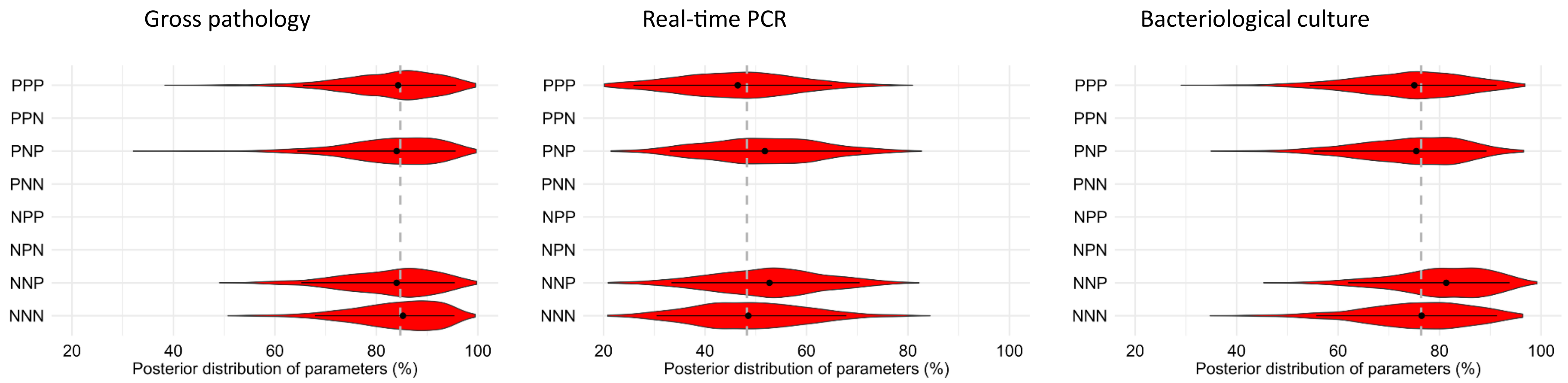

Cervid data removal from the pooled high prevalence group – cervid Sensitivity parameters in the high prevalence group

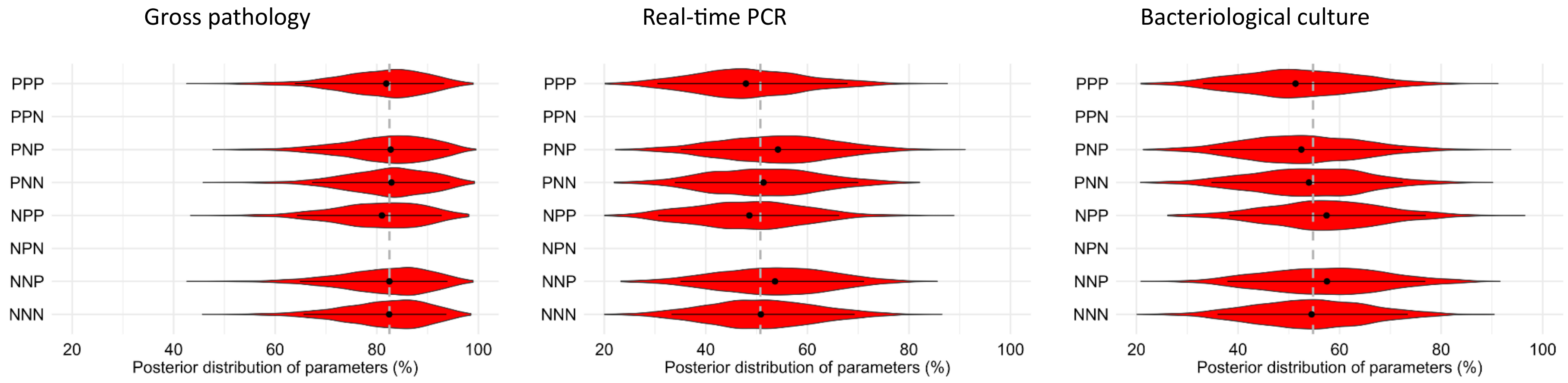

Model 3

Wild boar data removal – wild boar parameters

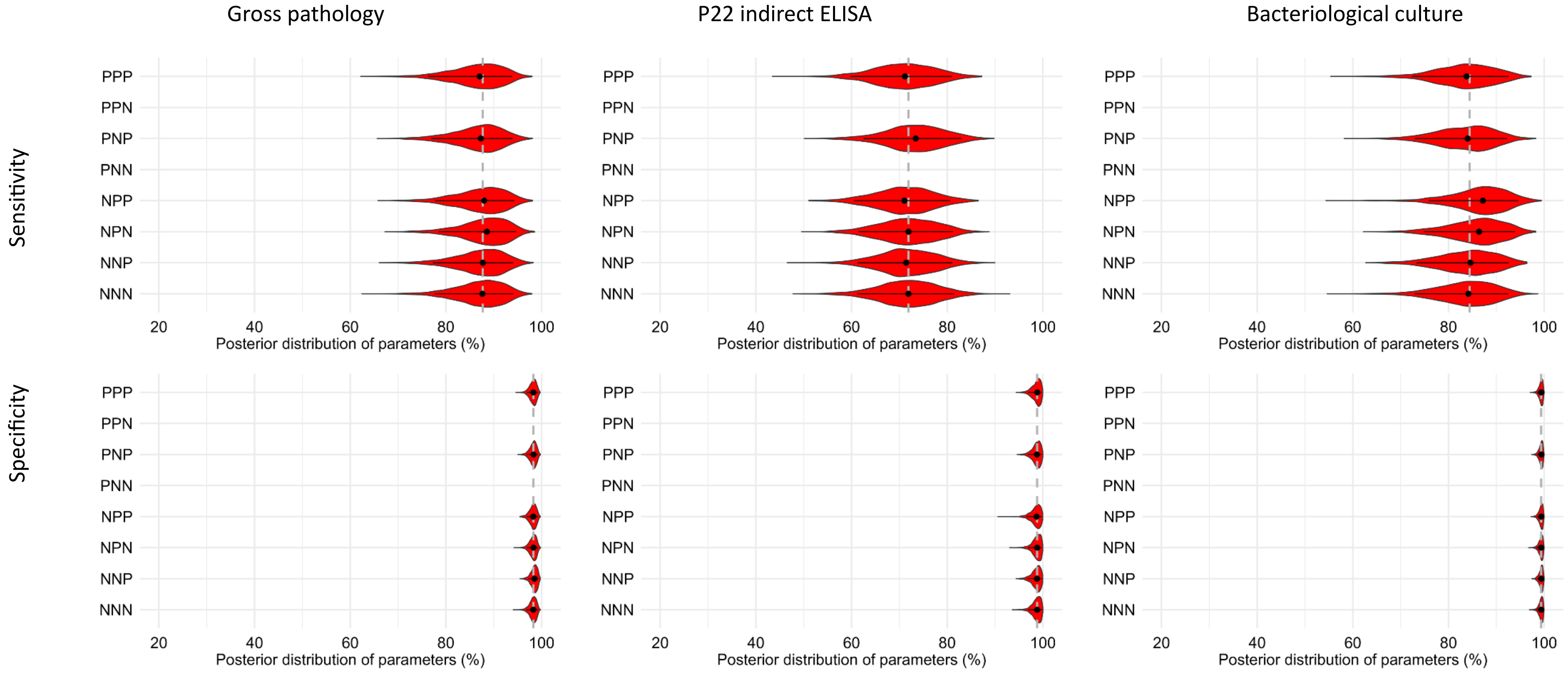

Model 3

Cervid data removal – cervid parameters

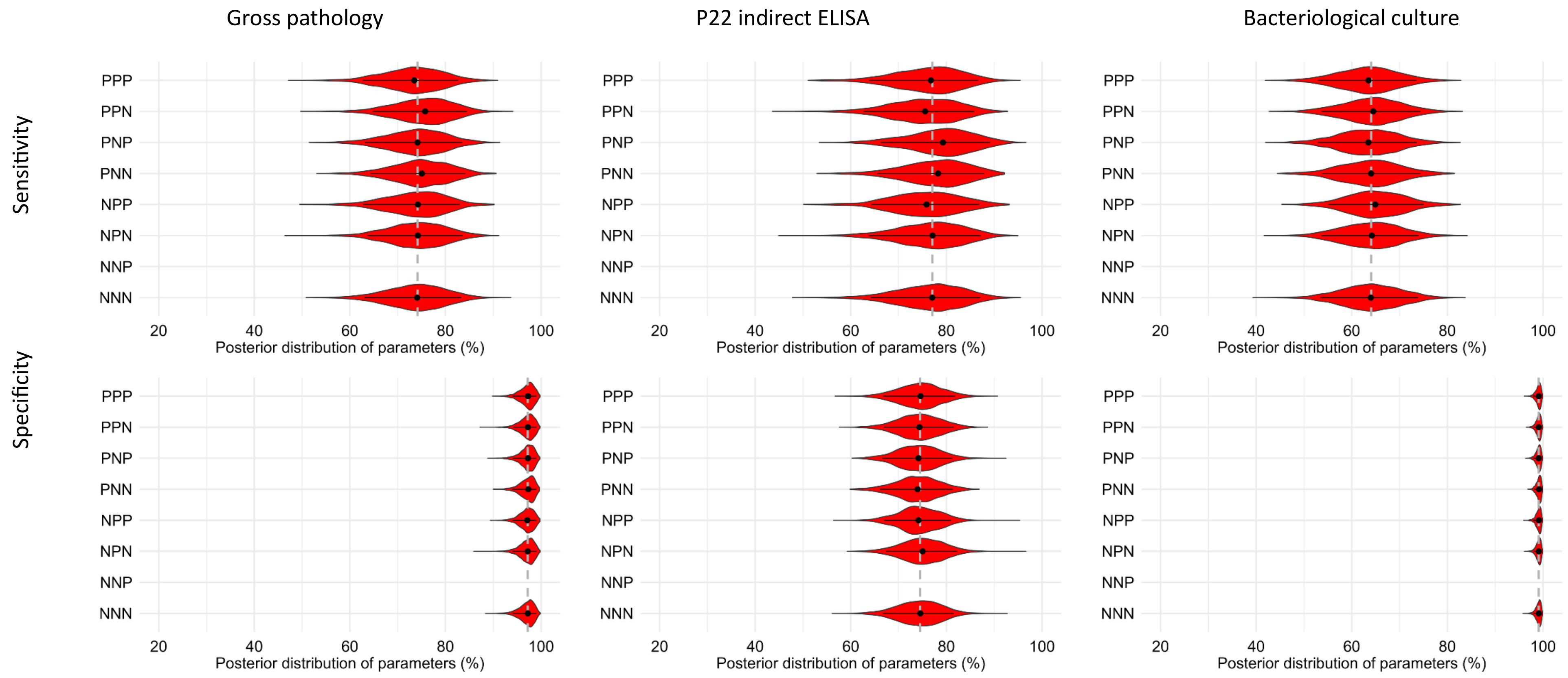

Supplement: Supplementary file 1 [file Data_Sheet_1.pdf]
